# The Azaindole Framework in the Design of Kinase Inhibitors

**DOI:** 10.3390/molecules191219935

**Published:** 2014-11-28

**Authors:** Jean-Yves Mérour, Frédéric Buron, Karen Plé, Pascal Bonnet, Sylvain Routier

**Affiliations:** Institut de Chimie Organique et Analytique (ICOA), Université d’Orléans, UMR CNRS 7311, Orléans F-45067, France; E-Mails: jean-yves.merour@univ-orleans.fr (J.-Y.M.); frederic.buron@univ-orleans.fr (F.B.); karen.ple@univ-orleans.fr (K.P.); pascal.bonnet@univ-orleans.fr (P.B.)

**Keywords:** azaindoles, kinase inhibitors, kinase binding mode, structural analysis

## Abstract

This review article illustrates the growing use of azaindole derivatives as kinase inhibitors and their contribution to drug discovery and innovation. The different protein kinases which have served as targets and the known molecules which have emerged from medicinal chemistry and Fragment-Based Drug Discovery (FBDD) programs are presented. The various synthetic routes used to access these compounds and the chemical pathways leading to their synthesis are also discussed. An analysis of their mode of binding based on X-ray crystallography data gives structural insights for the design of more potent and selective inhibitors.

## 1. Introduction

Azaindoles and their derivatives exhibit significant biological activities and the use of this framework has contributed to the generation of new therapeutic agents. The four azaindole positional isomers, which associate a pyridine and a pyrrole ring by a fused C-C bond, possess all of the criteria necessary to be excellent bioisosteres of the indole or purine systems. These three entities only differ by the substitution of a sp^2^ CH fragment by a sp^2^ nitrogen atom and *vice versa*.

Rare in Nature [1,2,3,4], azaindoles are interesting in terms of drug optimization strategies. Modification of Lipinski’s rule of five, solubility, pK_A_ and lipophilicity, target binding and ADME-tox properties can be modulated and finely tuned using the azaindole core instead of other bicyclic fused heterocycles [5,6,7,8,9,10,11,12,13,14]. Azaindoles have been recognized as privileged structures in biological process modulation, in medicinal chemistry and drug discovery programs [15,16,17,18,19,20]. Their commercial availability has steadily increased and synthetic innovation has been continuously updated. Specialized on-line research gates for chemical structures easily give a global view of the azaindole research domain with novel syntheses and unknown structures emerging all the time.

When considering the use of an azaindole scaffold instead of an indole one in active drugs, the 5-aza isomer first appears to be the most commonly encountered because of its strong homology with 5-hydroxy indoles, the main metabolites of indole which are present in several biomolecules (e.g., serotonine, melatonine, 5-HIA, 5-OHDPAT), but this is misleading. The most popular azaindole is indubitably the *N-*7 isomer which has generated more than 100,000 structures, the number of commercially available derivatives is twice that of all other isomers individually. Therefore it is also the most patented structure (Table 1). Reference Evolution from 2003 to 2013.

**Table 1:** Statistics for the azaindole framework in the chemical literature.

Azaindole framework	4-Azaindole	5-Azaindole	6-Azaindole	7-Azaindole
on-line substructure research ^a^	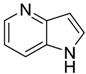	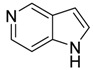	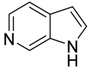	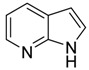
Scifinder				
chemical structures	16,505	13,632	34,762	100,384
commercially available	2517	2006	>2749	4273
References (patents)	2158 (1187)	2923 (961)	10,497 (1756)	6576 (2863)
Reference Evolution from 2003 to 2013	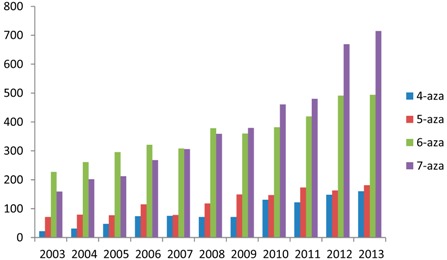			
Reaxys				
chemical structures	9701	8058	27,655	58,082
commercially available	707	639	1116	3503
References (patents)	583 (586)	724 (477)	3461 (890)	3951 (2026)
E molecules	128	150	138	2300
Ambinter	1105	1395	4132	>to 10,000

^a^: Search performed on 5 July 2014 using the azaindole framework as chemical substructure.

Writing an exhaustive review covering all of the biological activities of azaindole derivatives is virtually impossible. Several reviews exist which have compiled methods leading to these compounds which focused on specific biological activity [21,22,23,24]. To complete these reports, we have voluntary chosen a domain within our research area, the synthesis of kinases inhibitors. Since 2004, our group has been involved in the discovery of novel kinase inhibitors and has generated several strongly active and original series [25,26,27,28,29,30].

Among the various biological targets for azaindole derivatives, kinases are those of choice as the presence of the extra nitrogen atom increases the possible fits in the ATP active site which requires well positioned H bond donor acceptor systems [31]. Several azaindole derivatives have emerged from medicinal chemistry programs, and some of these have evolved into drug candidates for treating human disease.

The human kinome consists of more than 500 protein kinase members thereby making it one of the largest gene families. Protein kinases play a predominant regulatory role in nearly every aspect of cell biology. They regulate apoptosis, cell cycle progression, cytoskeletal rearrangement, differentiation, development, immune response, nervous system function and transcription. For these reasons dysregulation of protein kinases occurs in a variety of diseases including diabetes [32,33], inflammatory [34,35], cardiovascular [36,37,38] and nervous disorders [39,40]. Moreover, it is commonly accepted that protein kinases are excellent targets in oncology. Inhibiting cell cycle kinases has been largely explored in cancer research [41]. Novel approaches to the development of tyrosine kinase inhibitors and their role in the fight against cancer have been largely discussed in order to discover micro-targeted therapies [42]. Among the tyrosine-kinases observed in mitochondria Src kinases are the most important by modulating oxidative phosphorylation and apoptosis [43]. The aberrant growth of cancer cells is attributed to the dysregulation of cell cycle control and cell division. Nuclear kinases responsible for cell cycle progression, including the cyclin-dependant kinases (CDK), checkpoint kinases (CHKs), aurora kinases, polo-like kinases (PLKs) have all been investigated as drug targets [44]. The Erb B/HER protein kinases are among the most studied cell signaling families in biology. Downstream Erb B signaling modules include the phosphatidylinositol 3-kinase/AkT (PKB) pathway, the Ras/Raf/Met/Erk1/2 pathway and the phosphalipase C pathway [45]. PIM proteins belong to a family of ser/thr kinases composed of 3 members, PIM1-3 with greatly overlapping functions. PIM kinases are responsible for cell cycle regulation, anti-apoptotic activity and the homing and migration of receptor tyrosine kinases mediated via the JAK/STAT pathway. PIM kinases have also been found to be upregulated in many hematological malignancies and solid tumors. Enumeration of all of the kinases that are or will one day be a target in oncology is not an easy task. Their numbers increase yearly, and in many cases they are first linked to different pathologies.

To illustrate the extent to which azaindole derivatives have been used as kinase inhibitors and their contribution to drug discovery and innovation, we present herein the different protein kinases which have served as targets (in alphabetical order) and the known molecules which have emerged from medicinal chemistry and Fragment-Based Drug Discovery (FBDD) programs. Specifically developed chemistry methodology and full access routes are depicted. The common structural features which are essential to the mode of action of optimized drugs are presented. A binding mode analysis study which helps to better understand the interaction of azaindole heterocycles into the ATP binding site is also provided.

## 2. ALK Kinase Inhibitors

**Scheme 1:**
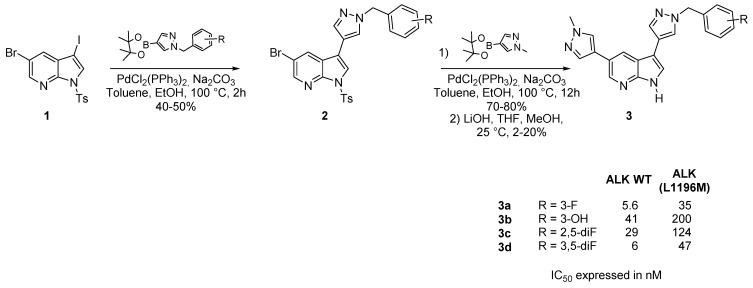
Preparation of several 3,5-disubstituted-7-azaindole derivatives.

Anaplasmic Lymphoma Kinase (ALK) is a transmembrane receptor tyrosine kinase (RTK) pharmacologically involved in brain development which exerts its effects on specific neurons in the nervous system. This kinase was then found to be a possible target in oncology, and several azaindoles were reported as good inhibitors [46,47]. 3,5-Disubstituted-7-azaindole derivatives **3** were readily prepared in two steps from 1-tosyl-3-iodo-5-bromo-7-azaindole (**1**) after two successive palladium catalyzed cross coupling reaction using 1-(substituted)benzyl- and 1-methyl- 3-boronate imidazoles. A first regioselective reaction occurred at the *C*-3 position with a second one at *C*-5 (Scheme 1). The tosyl protecting group was then removed in the presence of base, but this last step was characterized by very low yields. A docking study predicts the binding mode of these compounds in the kinase ATP active site [47].

## 3. Aurora Kinase Inhibitors

**Scheme 2:**
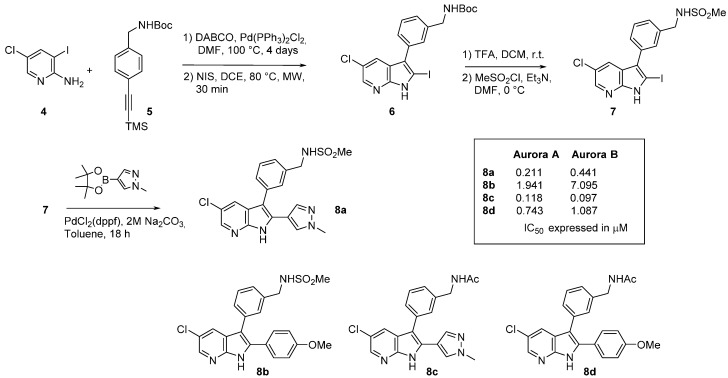
Synthesis of an Aurora inhibitor (8).

Aurora kinases have been studied as anticancer targets since their discovery in the 1990s [48]. This family is composed of three kinases designated Aurora A, B, and C which play key and distinct roles in the different stages of mitosis [49]. There have been many different types of active Aurora kinase inhibitors reported, and among these 7-azaindoles are valid competitors. In a recent article, formation of the azaindole scaffold was carried out by condensation of alkyne **5** with 5-chloro-3-iodo-2-aminopyridine (**4**) in the presence of DABCO and Pd(PPh_3_)_2_Cl_2_ (Scheme 2) [50]. Trimethylsilane (TMS) removal in the presence of NIS provided the 2-iodo derivative **6**. N-Boc deprotection followed by sulfonamide formation generated compound **7** which was submitted to Suzuki-Myaura cross-coupling reaction with het(aryl)boronate esters. Compound **8a** was obtained with an *N*-methyl pyrazole system in *C*-2. Three more compounds were thus produced following a similar strategy and tests revealed **8c** as the most active derivative.

**Scheme 3:**
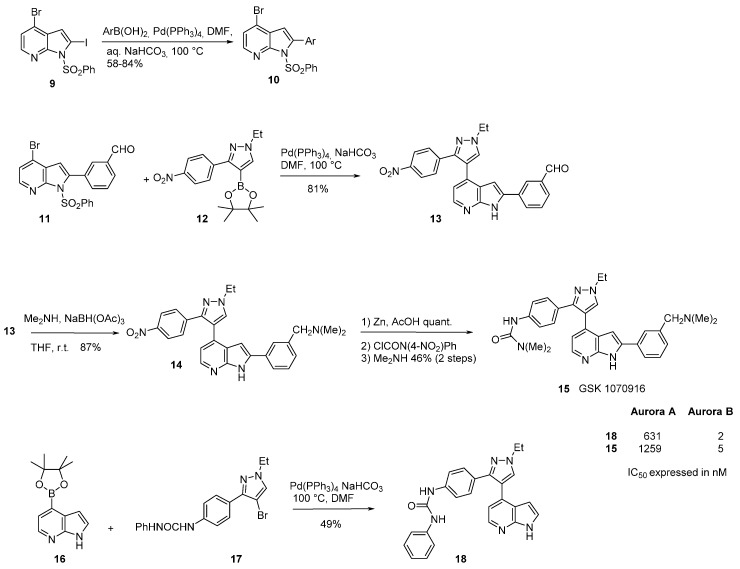
Synthesis of GSK 1070916.

In another example, a pyrazole scaffold was added to a 7-azaindole fragment in *C*-4 leading to the selective Aurora B/C inhibitor GSK 1070916 (**15**) [51,52]. The synthesis of this compound was based on the selective Suzuki cross coupling of 4-bromo-2-iodo-1-(phenylsulfonyl)-1*H*-pyrrolo-[2,3-b]pyridine (**9**) with diverse phenylboronic acids to give the 2-aryl intermediates **10** in good yields (Scheme 3). The synthesis of GSK 1070916 was carried out with 4-bromo-2-(3-formylphenyl)-1-phenylsulfonyl-1*H*-pyrrolo[2,3-b]pyridine (**11**) in a second coupling reaction with the *N*-ethylindazo boronate pinacolato ester (**12**) which gave the nitro derivative **13** in 81% yield. Reductive amination of the formyl group into compound **14** was achieved with dimethylamine and sodium triacetoxyborohydride in 87% yield. Reduction of the nitro group of **14** with zinc in acetic acid was followed by the introduction of the urea functionality to produce compound **15**. Interestingly, the first key Suzuki arylation was carried out selectively in position *C*-2 by displacement of the iodine atom *vs*. the bromine atom in *C*-4 of the azaindole moiety and this in spite of the steric hindrance induced by the azaindole protective group [53]. A truncated version of this molecule was also described. The synthesis of **18** was carried out from 4-boronate pinacolato ester-7-azaindole (**16**) which reacted with the functionalized bromo derivative **17** to give **18** in a 49% yield.

**Scheme 4:**
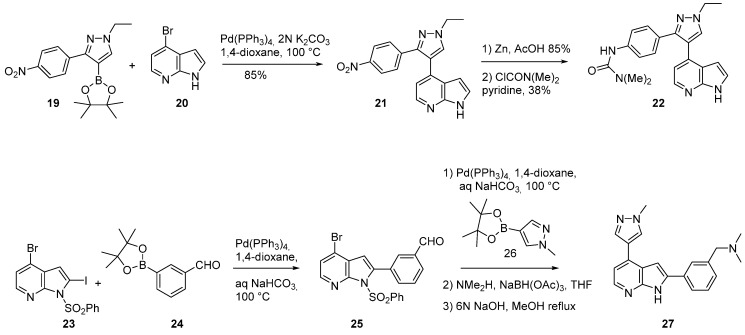
Synthesis of compounds **22** and **27**.

Compound **22** with no substituent in position *C*-2 of the 7-azaindole moiety and compound **27** with no urea chain on the pyrazole ring were prepared in order to formally establish the minimum pharmacophore of GSK1070916 (Scheme 4) [54]. Thus, 4-bromo-7-azaindole (**20**) and (1-ethyl-3-(4-nitrophenyl)pyrazol-4-yl)boronic pinacolato ester (**19**) were coupled in the presence of Pd(PPh_3_)_4_ and potassium carbonate to produce **21** in 85% yield. Reduction of the nitro group was achieved by zinc in acetic acid, followed by reaction of the amino group with dimethyl carbamoyl chloride to give the desired derivative **22**. (Scheme 4) [52]. Similarly, the synthesis of compound **27** was possible via two sequential Pd(PPh_3_)_4_ catalyzed coupling reactions followed by the functionalization of the aromatic aldehyde by reductive amination.

**Scheme 5:**
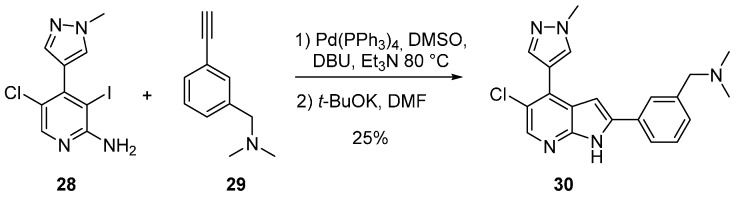
Synthesis of compound **30**.

The suppression of the arylurea moiety led to a disappearance in selectivity (**27**, IC_50_ Aurora A/Aurora B 128/5.7 nM). The authors speculated that substitution with a *C*-5 chloro atom on the aromatic ring could fill space in the binding pocket and/or break the co-planarity of the pyrazole and azaindole rings to more closely mimic the conformation of GSK1070916. From the 2-amino-3-iodo pyridine derivative **28**, a Sonogashira cross coupling reaction followed by a base induced annulation generated the final azaindole derivative **30** (Scheme 5 and Table 2) which could be considered as a fully selective dual Aurora A and B inhibitor, and an effective starting point for the development of another class of Aurora B inhibitors. 3D QSAR and molecular docking as well as cellular effects and fluorescence-activated cell sorting analysis were reported [55].

**Table 2:** Aurora A and B inhibition, IC_50_ values for compounds **22**, **27**, **30**, GSK1070916.

IC_50_ μM	22	27	30	GSK1070916
Aurora A	10,000	128	6.2	1100
Aurora B	26	5.7	0.51	3.2

## 4. Cdc7 Inhibitors

**Scheme 6:**
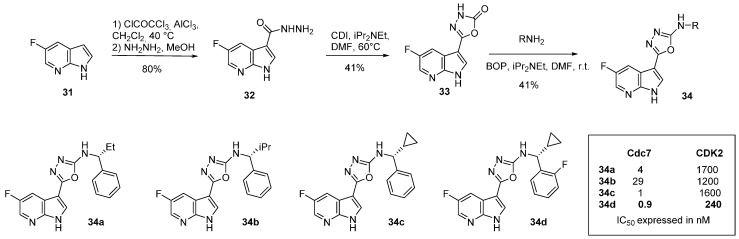
Synthesis of Cdc7 inhibitors **34**.

Cell division cycle 7 (Cdc 7) is a serine/threonine protein kinase involved in the initiation of DNA replication mainly in the S phase. Inhibitors of Cdc7 may be used as single agents or in combination with other chemotherapy [56]. Orally active 7-azaindole inhibitors of Cdc7 have been designed (Scheme 6). Starting from 5-fluoro-7-azaindole (**31**), an acylation at position *C*-3 with trichloroacetyl chloride followed by reaction with hydrazine, produced the hydrazide derivative **32** in 80% yield. Further reaction with 1,1'-carbonyldiimidazole led to compound **33** in 41% yield. This compound was condensed with different primary amines under peptide coupling conditions to produce a library of 12 derivatives of type **34**. The best inhibitor **34d** targeting the desired enzyme in the nanomolar range with a very high selectivity between CDK1, 2 and 9 (IC_50_ 240, 1600, 31 nM respectively). ADME-Tox data and cell effects have also been described [56].

**Scheme 7:**
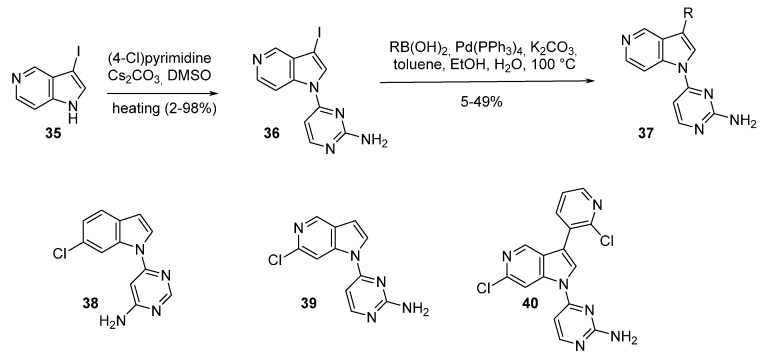
Synthesis of Cdc7 inhibitors **37**.

More recently, in the search for Cdc7 inhibitors with exhibited selectivity over the functionally related CDK2, the synthesis of various 5-azaindole derivatives was investigated (Scheme 7 and Table 3). An initial High Throughput Screening (HTS) identified the indole **38** with a 6-chloro substituent as having good selectivity for Cdc7 over CDK2. 3-Iodo-5-azaindole (**35**) was first *N*-arylated with 4-chloropyrimidine by a S*_N_*Ar reaction to give compound **36**. Suzuki coupling with various aryl or heteroaryl boronic acids in the presence of Pd(PPh_3_)_4_ afforded **37** in 5%–49% yields. The authors then developed substituted 5-azaindole derivatives **39** and **40** which were unfortunately not as active. Other isomeric 4-, 6-, 7- azaindoles showed lower inhibition activity/selectivity and did not improve metabolic stability [57]. It was speculated that the loss in potency may have been due to the hinge binder pyrimidine being orientated out of the preferred binding conformation due to an intramolecular nitrogen/nitrogen azaindole/pyrimidine repulsion. A dihedral study predicted the potency of inhibitors which required a planar as well as a *syn* conformation [57].

**Table 3:** SAR of Cdc7 inhibitors **37**–**40**.

Compound	R	IC_50_ Cdc7 (μM)	IC_50_ CDK2 (μM)	Compound	IC_50_ Cdc7 (μM)	IC_50_ CDK2 (μM)
**37a**	H	0.98	3.7	**38**	0.066	3.7
**37b**	C_6_H_4_	0.10	0.31	**39**	0.66	38
**37c**	2-ClC_6_H_4_	0.011	0.11	**40**	0.03	>80
**37d**	3-ClC_6_H_4_	0.16	>83			
**37e**	4-ClC_6_H_4_	0.33	0.06			
**37f**	3-pyridinyl	0.16	1.1			
**37g**	3-(2-Clpyridinyl)	0.007	0.31			
**37h**	4-pyridinyl	0.25	0.16			
**37i**	3-furanyl	0.12	0.10			
**37j**	3-pyrazole	0.58	0.55			

**Scheme 8:**
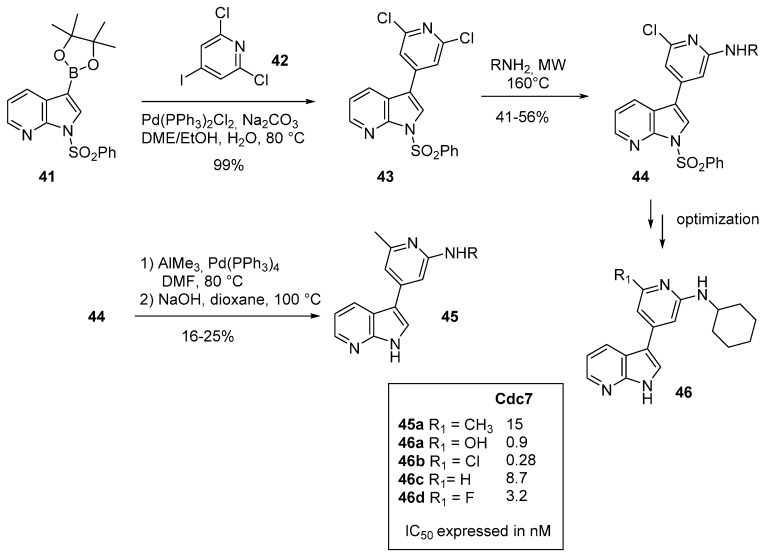
Synthesis of Cdc7 inhibitors **45**.

Recently, Tong and Steward have also reported their optimization process which successfully led to Cdc7 inhibitors having a 7-azaindole core [58]. Lack of substitution on the pyrrole nitrogen atom seemed to be essential for enhanced activity. The 7-azaindole-3-boronate (**41**) was reacted with the 4-iodo-2,6-dichloropyridine (**42**) to produce **43** in 99% yield (Scheme 8). Various primary amines such as cyclohexylamine, 4-hydroxycyclohexylamine and benzyl amine were then reacted under microwave irradiation at 160 °C to furnish compounds **44** in moderate yields (41%–56%). Introduction of a methyl group with trimethyl aluminium was achieved in the presence of Pd(PPh_3_)_4_. Cleavage of the benzenesulfonyl group in basic media gave the straightforward synthesis of compounds of type **45** in low yields (16%–25%). No 7-azaindole modification was done, and other derivatives which completed the library were focused on the modulation of the *C-3* pyridine fraction. Fluorine atom, cyano, hydroxyl and primary amide were used, and the best inhibition of Cdc7 occurred with cyclopropyl amine and hydroxyl pyridine substituents. Attempts to crystallize Cdc7 have not been successful, and structural information has been obtained from ligand docking into homology models of Cdc7 kinases or crystallographic studies of ligand-bound surrogate kinases such as PIM or GSK3β. The binding mode was clarified by a co-crystalline X-ray structure of **50** (X = Cl) in a surrogate protein GSK3β confirming the 7-azaindole moiety of the molecule as the hinge-binding motif with the pyridine pointed toward the conserved Lys residue. The twist of the pyridine ring relative to the azaindole is flatter, at about 10° of that expected for a bis aryl system. Replacing the chlorine atom with H or Me led to weaker hydrophobic interactions with Met 134 and Val 195 resulting in reduced Cdc7 inhibition.

**Scheme 9:**
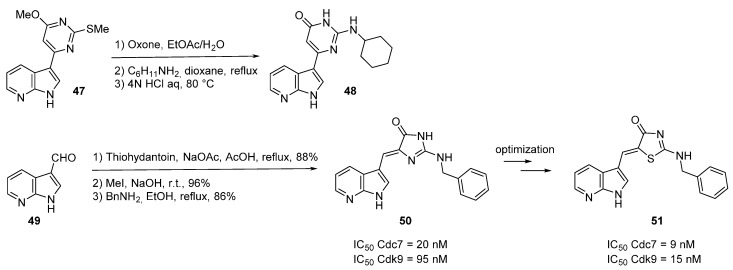
Synthesis of Cdc7 inhibitors **48** and **50**.

Similarly, compound **48** was prepared by displacement of the SO_2_Me group of compound **47** with cyclohexylamine in refluxing dioxane, followed by generation of the pyrimidone in acidic medium (Scheme 9). Addition of one nitrogen atom is well tolerated. Compound **47** binds to Cdc7 and exhibited a Ki value of 0.07 nM [59]. 1*H*-Pyrrolo[2,3-*b*]pyridine derivatives have also been identified as Cdc7 inhibitors [60]. 7-Azaindolylideneimidazoles **50** are easily prepared by condensation of thiohydantoin with 7-azaindole-3-carboxaldehyde (**49**). Compound **50** with a *C-2* NH-benzyl substituent of the imidazolone motif had an IC_50_ value of 20 nM and was more active than the derivative with a *C-2* phenyl. An *E* or *Z* configuration of the double bond influenced the binding mode with the kinase with the Z isomer leading to better interactions. Bioisosteric nitrogen *vs.* sulfur atom also led to interesting results, the thio derivative **51** was the best active drug (IC_50_ Cdc7 = 9 nM) but with a decreased selectivity towards CDK9 (IC_50_ = 15 nM).

## 5. Check-Point Kinase (CHK1) Inhibitors

**Scheme 10:**
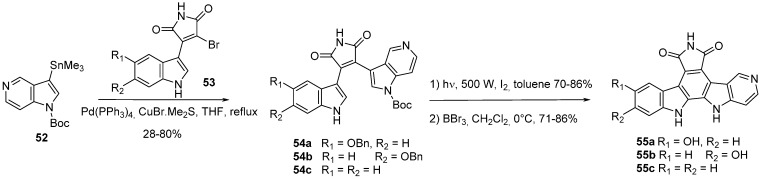
Synthesis of CHK1 inhibitors **55**.

Check-point kinase 1 (CHK1) is a serine/threonine kinase occupying a central position in cell regulation and DNA repair mechanisms. We have reported the synthesis of 5-azaindolocarbazoles **55** as cytotoxic agents and Chk1 inhibitors [29]. Stille reaction of 1-Boc-3-trimethylstannyl-5-azaindole (**52**) with indole bromomaleimides **53** produced the 3-(5-azaindolyl)-5-indolyl maleimides **54** in 28%–80% yields (Scheme 10). Cyclization to give the carbazole framework **55** was achieved by ultraviolet irradiation in the presence of iodine in good yields (70%–86%). Benzyl group removal was possible with an excess of boron tribromide in dichloromethane at 0 °C. The presence of hydroxy substituents on **55** gives more active derivatives than unsubstituted ones. Compound **55b** is the most potent against Chk1 (IC_50_ = 14 nM), the absence of substitution on the indole ring (R_1_ = R_2_ = H) decreased the activity to the micromolar range (**55c**, Chk IC_50_ = 1.5 μM).

## 6. C-Met Kinase Inhibitors

**Scheme 11:**
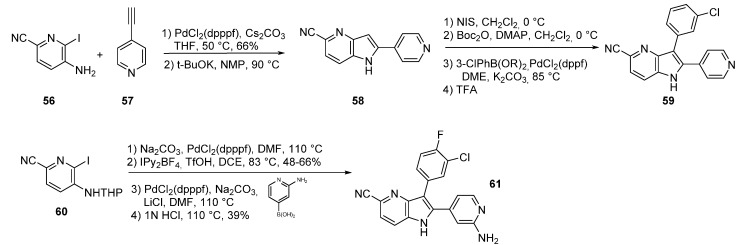
Synthesis of c-Met kinase inhibitors **59** and **61**.

C-Met kinase is a receptor tyrosine kinase present in both normal and malignant cells. This enzyme promotes signaling pathway activation and is involved in tissue regeneration, angiogenesis, and enhanced cell motility. In a variety of cancer types c-Met is overexpressed and leads to high proliferation scattering, invasiveness and metastasis development. ATP competitive c-Met kinase inhibitors such as compound **59** have been reported in the literature [61]. A Sonogashira reaction of 4-pyridinylethyne (**57**) with iodopyridine **56** afforded the 5-cyano-2-(4-pyridinyl)-4-azaindole derivative **58** after cyclization with potassium *tert*-butoxide (Scheme 11). Iodination at position *C*-3 was effective with NIS. Boc protection of the nitrogen atom was followed by a Suzuki coupling and treatment with trifluoroacetic acid to give the 2,3-diaryl-7-azaindole **59**. Alternatively, a sequence with THP protected anilines led to *C*-2 substituted pyridine derivatives such as **60**. Only a few derivatives were active on c-Met, the best ones inhibited the kinase at IC_50_ = 40 nM (compound **59**) and 130 nM (compound **61**). It is noteworthy that small structural modifications give a strong range of activity.

**Scheme 12:**
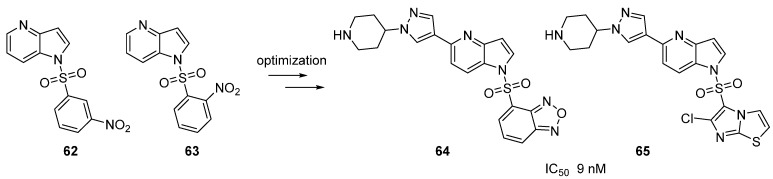
C-Met kinase inhibitors **62**–**65** following lead optimization.

Two *N*-nitrobenzenesulfonyl-4-azaindoles derivatives **62** and **63** were identified as c-Met inhibitors with an IC_50_ of 70 and 20 nM respectively [62]. In this article, *C*-3 sulfur and sulfoxide azaindoles were first developed as part of a medicinal chemistry program but the most promising series remained the *N*-1 substituted scaffold. Within that family, further optimization showed that substitution at the 6 position of the azaindole ring was possible and that a piperazine group gave the best results. Attempts were then made to isosterically replace the nitrophenyl moiety by preparation of benzofurazan and chloroimidazo[2.1-*b*]thiazole analogues **64** and **65** (Scheme 12). Activities were significantly enhanced with an IC_50_ of 9 nM for both molecules. Docking of **64** with c-Met kinase showed possible interactions of the imidazothiazole motif with Asp 1222 and Tyr1230. Unfortunately methods leading to final derivatives were poorly described.

**Scheme 13:**
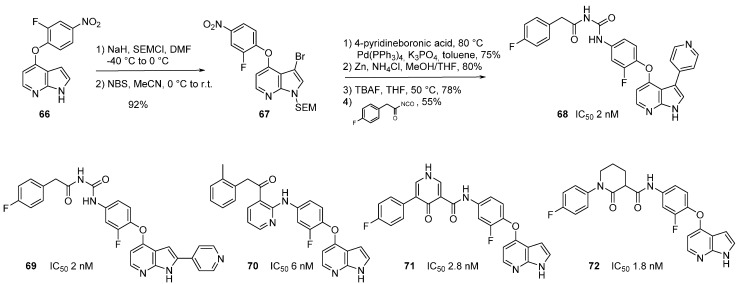
Synthesis of c-Met kinase inhibitor **68** and analogues.

To increase the binding site affinity and the probability of establishing hydrogen bonds with the two nitrogen atoms, workers at Bristol-Myers Squibb selected the 7-azaindole scaffold to design novel c-Met inhibitors [63]. 4-(2-Fluoro-4-nitrophenoxy)-7-azaindole (**66**) was SEM protected then brominated with NBS to give **67** in 92% yield. Introduction of a pyridinyl group at position *C*-3 was performed with the corresponding boronic acid via a Suzuki cross-coupling reaction. Reduction of the nitro group with zinc, followed by cleavage of the SEM group and ultimate urea formation led to compound **68** in 24% total yield (Scheme 13). This compound had an IC_50_ value of 2 nM, its positional isomer **69** with the pyridinyl substituent in position *C*-2 showed exactly the same potency against c-Met kinase. Both **68** and **69** occupy the ATP binding site where the protein is in an inactive conformation, *i.e.*, the activation loop folds back toward the ATP-binding pocket. The *N*-7 atom accepts an H-bond from the hinge region backbone NH of Met 1160. The *N*-1 atom donates an H-bond to the carbonyl of Met 1160. The *C*-3 position of the 7-azaindole motif points toward the ribose pocket and the *C*-2 position lead to the surface-exposed extended hinge region. Derivatives **70**–**72** were described to demonstrate the “druggability” of the series giving several molecules acting in the nanomolar range [64,65].

## 7. DYRK1A Kinase Inhibitors

**Scheme 14:**
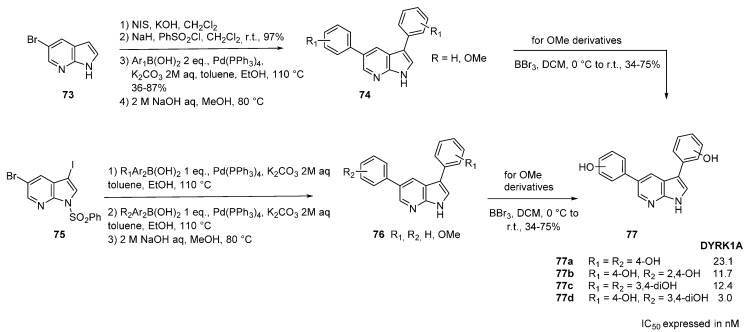
Synthesis of DYRK1A inhibitors **77**.

Dual-specificity tyrosine phosphorylation-regulated kinase 1a (DYRK1A) belongs to the DYRK subfamily of protein kinases which is present in human fetuses and brain. Along with cyclin-dependent kinases (CDKs), mitogen-activated protein kinases (MAPKs), glycogen synthase kinases (GSKs), and CDK-like kinases (CLKs), the DYRK family is part of the CMGC group. Gene encoding for DYRK1A is located on chromosome 21, overexpression is well characterized and this target has been validated for several pathologies such as Downs syndrome, Alzheimer’s disease as well as in oncology [66,67,68,69,70,71,72,73,74,75]. 6- and 7-Azaindole derivatives have been specifically developed as DYRK1A inhibitors [76]. For the preparation of the 3,5-diarylated-7-azaindoles a standard synthetic route was used (Scheme 14). The 5-bromo-7-azaindole (**73**) was iodinated at position 3 with NIS then *N*-protected with a benzenesulfonyl group in almost quantitative yield. Symmetrical di-arylated compounds **74** were obtained using 2 equivalents of an arylboronic acid. Unsymmetrical 3,5-diarylated derivatives **76** were obtained by two successive Suzuki-Miyaura cross-coupling reactions starting from compound **75**. Deprotection of the nitrogen atom of **74** and **76** in basic medium was followed by de-*O*-methylation with BBr_3_ (3 equivalents per methyl group) producing the hydroxyl derivatives **77**. Docking studies at the ATP binding site indicated multiple H-bond interactions with the peptide backbone (Glu 239, Leu 241) for the 7-azaindole core while hydroxyl substituents developed interactions with Lys 188 and Ileu 165. Hydroxyl derivatives were more active than the corresponding methoxy ones. Synthesis in the 6-azaindole series was also performed but the derivatives were considerably less active than the 7-azaindole ones. Interestingly, when these derivatives were tested against a representative kinase panel a relative selectivity appeared with compounds acting mainly on the DYRK1A family.

**Scheme 15:**
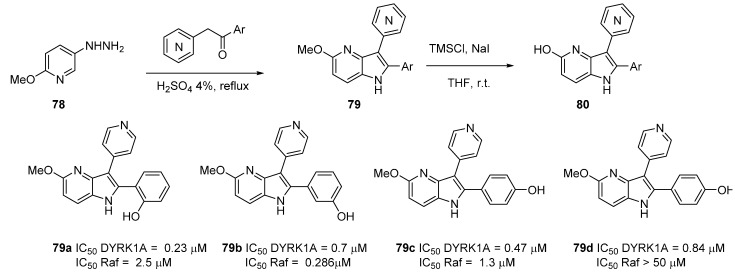
Synthesis of DYRK1A inhibitors **79** and **80**.

Our group has successfully developed DYRK1A inhibitors with several original heterocyclic cores including 4-azaindoles [28,77]. These syntheses were carried out by a novel Fischer reaction whose main advantage is the synthesis of the functionalized 4-azaindole building block in one step. Starting from 5-hydrazinyl-2-methoxypyridine (**78**) the condensation of pyridine acetophenones in acidic media conveniently furnished the derivatives **79** (Scheme 15). Methoxy removal was possible with *in situ* formed TMSI. The final compounds were evaluated on a panel of 5 kinases in order to evaluate their selectivity and on 7 cancer cell lines to determine their *in cellulo* potency and cytotoxic effects. Only c-Raf and DYRK1A inhibitions were found. Docking studies fully explained these results and SAR indicated that the presence of the methoxy group mainly afforded DYRK1A inhibitors, the best being **79d**, while the corresponding hydroxyl substitution **80** only gave c-Raf inhibition.

**Scheme 16:**
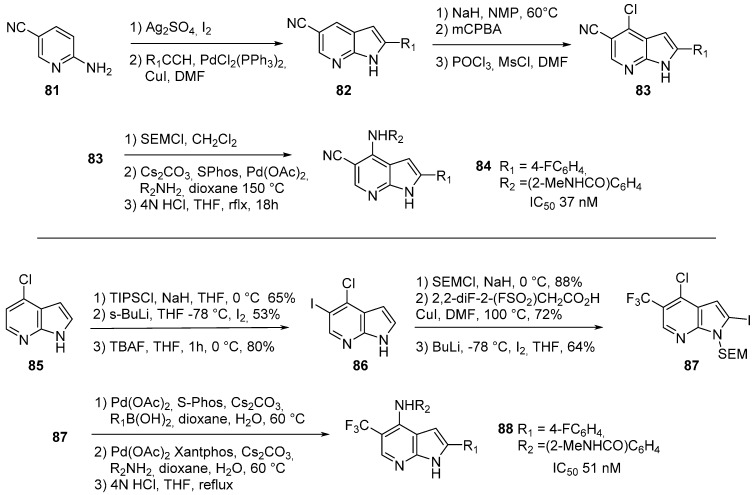
Synthesis of Focal adhesion kinase inhibitors.

## 8. FAK Inhibitors

Focal adhesion kinase (FAK) is a non-receptor tyrosine kinase that modulates cell adhesion, migration, proliferation and survival in answer to extracellular stimuli. To date, FAK is considered as an antineoplastic target as these inhibitors induce apoptosis and sensitize tumor cells to chemotherapy. Several interesting compounds were recently reported as part of a fragment-based discovery program [78]. Two different synthetic strategies were used to prepare the desired derivatives: construction of the bicyclic azaindole core using commercially available 2-aminopyridines, or successive selective functionalization of the 7-azaindole template (Scheme 16). Thus, the 5-cyano analog **84** was synthesized from the 4-cyanoaniline by iodation, followed by a Sonogashira cross coupling and annulation in basic media. An oxidation of the azaindole pyridine ring and treatment with POCl_3_ led to the chloro derivative **83**. The final steps included SEM protection, Hartwig-Buchwald amination and removal of the protective group to afford **84** which has an IC_50_ value of 37 nM. In the second sequence, chloro-7-azaindole **85** was protected with the bulky protecting group TIPS (to prevent lithiation at position *C*-2), then *ortho-*lithiation afforded the 5-iodo-4-chloro-7-azaindole (**86**) after *N*-deprotection with tetrabutylammonium fluoride. Compound **86** was protected with a SEM group and introduction of a trifluoromethyl group at position *C*-5 was achieved with 2,2-diF-2-(FSO_2_)CH_2_CO_2_H in the presence of CuI in 72% yield. Iodination in position *C*-2 with BuLi/I_2_ at −78 °C afforded compound **87** in 64% yield. Introduction of a phenyl group at position *C-2* was realized in the presence of palladium acetate and S-Phos and the corresponding aryl boronic acid. A Hartwig-Buchwald amination with various amines was then achieved in position 4 with catalytic palladium acetate and Xantphos, followed by SEM deprotection in acidic media to afford compound **88** which has an an IC_50_ value of 51 nM.

## 9. IKK2 Inhibitors

**Scheme 17:**
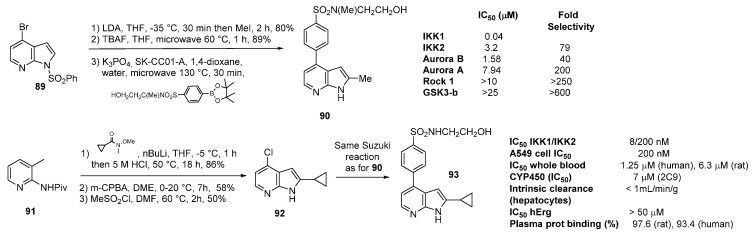
Synthesis of IKK2 inhibitors.

Two IKK isoforms 1 and 2 are known. IκK kinase (IKK) is a serine threonine protein kinase located in the cytoplasm which destabilizes the IκK/NFκB complex by phosphorylation and regulates the mitotic kinase Aurora A. The direct consequence is the validation of the IKK family as crucial targets in inflammatory (rheumatoid arthritis, COPD and asthma), and autoimmune disorders as well as in cancer via the regulation of the cell cycle. In this area, ATP-competitive 4-[4-(alkylsulfonamido)-aryl]-7-azaindoles represent a novel class of IKK2 inhibitors. A first lead was obtained after three routine steps with a regioselective directed metallation in *C*-2, deprotection and a palladium catalyzed Suzuki reaction (Scheme 17). Innovation came from the nature of the boron ester used [79]. Derivative **90** presented good IKK2 potency, and an impressive almost 80 fold selectivity over IKK1 was observed. The selectivity profile of **90** was also determined against a panel of 36 kinases. Only two kinases (IKK1 and AurB) were inhibited within 100-fold of IKK2.

A docking model was generated to explain the binding mode and SAR was established by several pharmacophore modifications mainly realized on the sulfonylamide residue and in position *C*-2 of the 7-azaindole. Further optimization gave the cyclopropyl derivative **93**, which was synthesized in four steps from the picoline derivative **91**, and which was the most active IKK2 inhibitor [80]. Additionally, full *in vitro* and physicochemical profiles were favorable (good selectivity and pharmacokinetics, oral bioavailability, low metabolism). *In vivo* effects on inflammation were also determined.

**Scheme 18:**
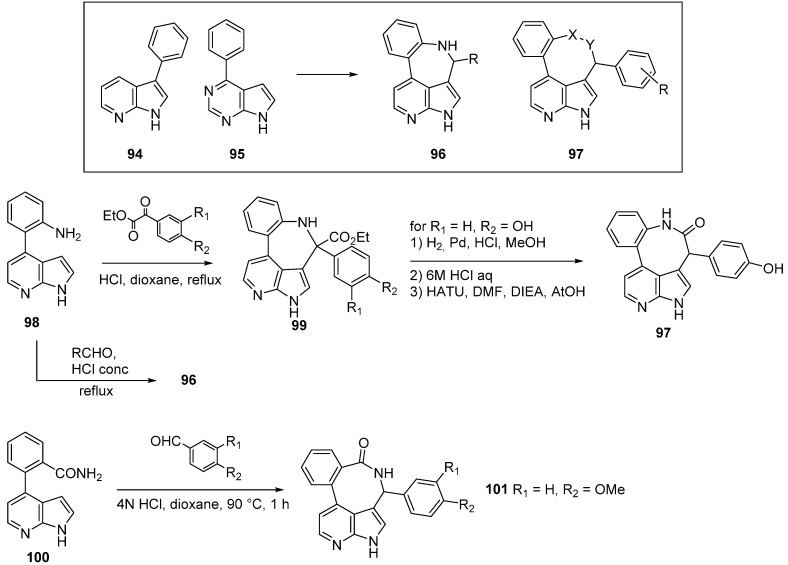
Synthesis of JAK kinase inhibitors.

## 10. JAK2 Inhibitors

The Janus kinases or JAKs are a family of intracellular tyrosine kinases that are implicated in the signaling process of many cytokine receptors and more particularly in those involved in the pathogenesis of inflammatory diseases [34]. There are four known mammalian intracellular non-receptor tyrosine kinases in the Janus kinase family (JAK1-3, TYK2). In 2005 it was reported that the occurrence of a single residue mutation in JAK2 was commonly found in patients diagnosed with myeloproliferative disorders [81,82,83,84,85,86]. Blocking the kinase activity of JAK2 by targeting the ATP binding site thus became an attractive therapeutic target [87,88]. Two molecules, the *C*-3 aryl-7-azaindole derivative **94** (IC_50_ JAK2 260 nM) and the aryl purine derivative **95** (IC_50_ JAK2 496 nM) emerged as low JAK inhibitors (Scheme 18). Overlaying the putative hinge binding orientations of the two hits, the authors suggested the possibility of increasing inhibitory activity by incorporating a bridge and performing a ring closure. More constrained and rigid compounds with a 3,4-fused seven- or eight-membered central ring such as **96** and **97** were designed. For example compounds **97** with an eight-membered central ring were prepared from 4-(phenyl)-7-azaindole (**98**) and **100**. Pictet-Spingler condensation with pyruvate ester provided the cyclized compound **99**. *N*-benzyl hydrogenation, followed by ester hydrolysis and intramolecular lactamization produced compound **97** as a mixture of atropoisomers. A simple acidic aldehyde condensation with **98** gave a large library of derivatives of type **96** whereas starting from **100** the retroamide **101** was produced. The co-crystalline structure of JAK2 and one azaindole derivative was obtained. The inhibitor forms two hinge H bond with the azaindole moiety to the protein through Leu 932 and Glu 930. An additional phenolic OH is a dual H-bond acceptor with the carboxylate of Glu 898 and H-bond donor with Phe 995. Strong inhibition was observed with compounds of type **96**. Derivative **97** (IC_50_ JAK2 1 nM, JAK3 5 nM) for which all pharmacokinetic parameters were determined, was used orally in an *in vivo* leukemia assay. Treatment was highly effective as administration of the JAK inhibitor prolonged animal survival for 32 days. These reports open the way to macrocyclic azaindole kinase inhibitors.

**Scheme 19:**
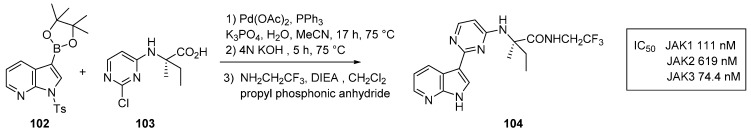
Synthesis of decernotinib (**104**).

Decernotinib (**104**) is a JAK inhibitor in development by Vertex, and is currently in phase 2 clinical trials for rheumatoid arthritis [89,90]. The short synthesis of decernotinib involved the 7-azaindole boronate ester **102** which was reacted with the chloropyrimidine derivative **103** in a Suzuki cross-coupling reaction (Scheme 19). Amidification of the resulting acid with 2,2,2-trifluoroethanamine afforded **104**.

## 11. KIT/FMS Dual Kinase Inhibitors

As mono-targeted therapy has sometimes led to disappointing results and requires the simultaneously use of several kinase inhibitors, another strategy has emerged, the concept of dual inhibitors. *In silico* drug design reaches its full potential in the development of these types of compounds as seen in a recent article concerning KIT/FMS kinase inhibitors [91]. Agents that target microenvironment modifications and tumor cell growth can lead to drugs for two main interdependent pathologies which are oncology and inflammation. In this context, KIT (v-kit Hardy-Zuckerman 4 feline sarcoma viral oncogene homolog) and FMS (McDonough feline sarcoma viral (v-fms) oncogene homolog) kinases offer multiple potential therapeutic opportunities to control inflammation (rheumatoid arthritis) as well as cancer progression. In the aforementioned article, FMS inhibition was expected to reduce monocyte maturation and osteoclastogenesis whereas KIT inhibition was expected to induce mast-cell apoptosis, thereby reducing the production of inflammatory cytokines and degradative molecules in the synovium. Several azaindole derivatives were designed in order to find a new series of molecules which could fulfill a key hydrogen bond interaction seen in the co-crystallization of PLX070 (**105**) with fibroblast growth factor receptor 1 (FGFR1).

**Scheme 20:**
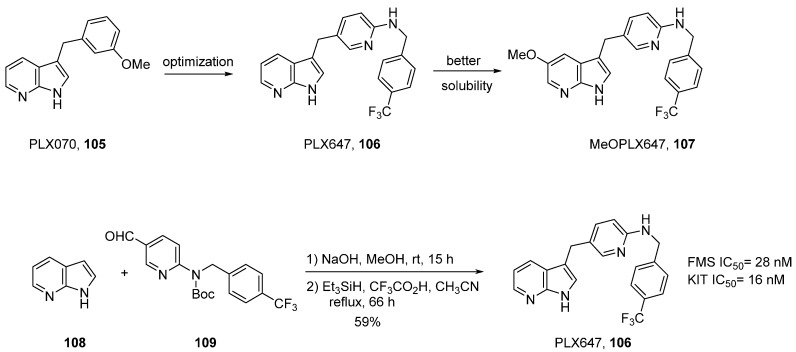
Development and synthesis of a dual KIT/FMS kinase inhibitor.

PLX647 (**106**) was easily obtained via the condensation in position *C*-3 of aldehyde **109** and 7-azaindole **108** (Scheme 20). Reduction of the created secondary alcohol with triethylsilane in acidic medium gave the final compound in a global yield of 59%. This compound was shown to be a highly specific dual KIT/FMS kinase inhibitor. It was tested against a panel of 400 kinases at a concentration of 1 μM, 35 fold above its enzymatic FMS IC_50_ and 60 fold above its KIT enzymatic IC_50_. Only nine kinases were inhibited by more than 50%. This compound also proved to be efficient in FMS and KIT expressing cell lines, (M-NFS60 cell IC_50_ 0.38 μM; M-07 cell IC_50_ 0.23 μM). In addition PLX647 blocked the activation of macrophages, osteoclasts and mast cells controlled by KIT and FMS kinases and its efficiency in rodent model was proven.

## 12. PAK1 Kinase Inhibitors

**Scheme 21:**
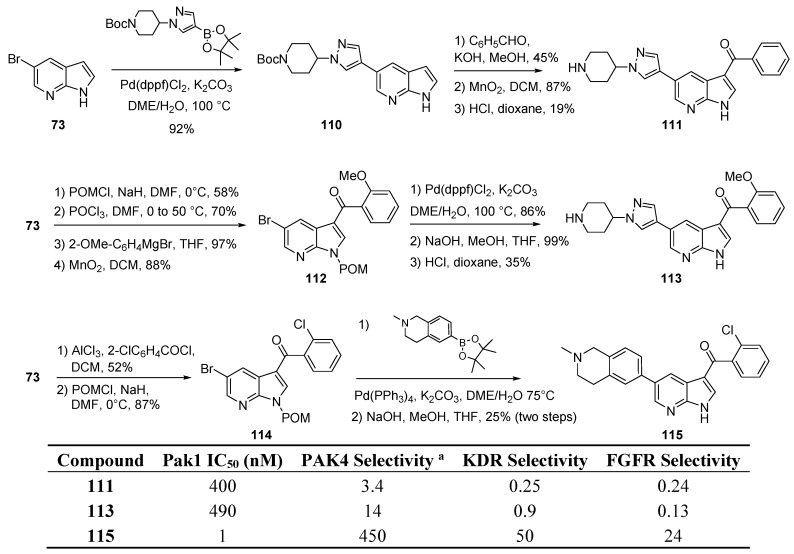
Synthesis of PAK1 inhibitors.

^a^ Selectivity calculated from the ratio of IC_50_ values.

The p21 activated kinases of type PAK are serine-threonine specific kinases. In particular PAK1 is reported to coordinate insulin actin remodeling and glucose uptake, and reduce injury after tissue traumatism [92,93]. Inhibition of this enzyme is important in limiting cancer progression and cell adhesion. PAK1 is linked to several signaling pathways as well as to Rho GTPases, enzymes involved in adhesion and growth-factor activated molecular switches. When activated, Rho-family GTPases transmit signals by recruiting a variety of effector proteins, including the protein kinases PAK [94]. Only a few inhibitors of PAK1 have been reported. Very recently, the use of the 7-azaindole motif was described in the literature with improved potency and selectivity for PAK inhibition [95]. The crystal structure of the best inhibitor in the active domain site was furnished and proved that the azaindole core binds efficiently with the hinge region and establishes strong interactions with Glu 345 and Leu 347 residues. Selectivity was provided by occupying the specific pocket as well as binding to gatekeeper residues. A wide optimization study accompanied the SAR studies. 3-Ketoaryl-5-aryl-7-azaindole derivatives **111** and **113** were modulated to afford lead compound **115** with an inhibition in the nanomolar range on PAK1 activity and with high selectivity toward PAK4, KDR and FGFR1 (Scheme 21). Cell quantification of pPAK in cells was measured to elucidate the action mechanism and some ADME parameters were measured. Derivatives were elaborated from the 5-bromo-7-azainindole (**73**). A Friedel-Crafts acylation led to substitution in C-3 whereas a Suzuki-Miyaura cross coupling reaction, which required the early preparation of boronic esters led to arylation in *C-5*. Direct arylations in *C-5* were achieved without any indole protection whereas all reaction performed on 3-carboxylated azaindoles required the transitory used of a pivaloyloxymethyl (POM) group.

## 13. p38α MAP Kinase Inhibitors

**Scheme 22:**
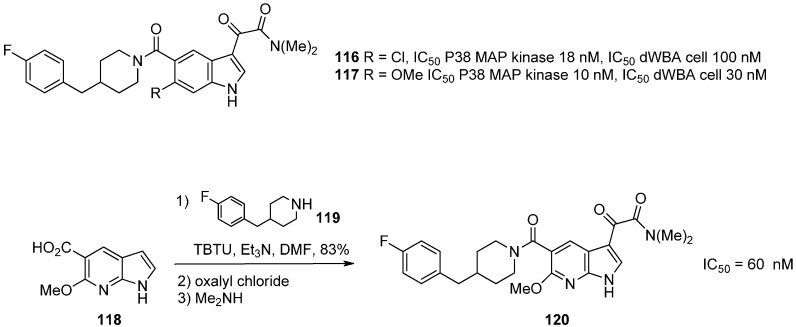
Synthesis of MAP kinase inhibitors.

There are three main classes of proline-directed serine-threonine MAP (mitogen-activated protein) kinases. They are activated by dual kinases termed MAP kinase kinases (MKKs) by phosphorylation of a tyrosine in the loop proximal to the ATP and substrate binding sites. In this family, p38 MAP kinase regulates cytokine biosynthesis involved in inflammation (proinflammatory cytokine interleukin-1β). and tumor necrosis (actor-α TNFα). The binding mode of the main p38 inhibitors is known [96,97]. Inhibitors of p38α MAP kinase useful in inflammatory disease have been synthesized using an azaindole skeleton. In a first study, 4-fluorobenzylpiperidine indole-based p38α MAP kinase inhibitors **116** and **117** were reported with good activity (Scheme 22) [98]. The indole derivatives were docked in the ATP binding site and their proposed interaction mode indicated a critical hydrogen bond with the kinase backbone at hinge amino acid Met-109. Additionally, the 4-fluorobenzyl group occupied an adjacent hydrophobic pocket leading to activity and selectivity. The presence of the 6-chloro or -OMe indole substitution further improved p38 enzymatic potency. Substituting other heterocycles for the indole was then performed. Compound **120** was obtained by condensation of the piperidine intermediate **119** with acid **118**, followed by the reaction of oxalyl chloride at the *C*-3 position of the 7-azaindole and terminal amidification with dimethylamine (Scheme 22). Derivative (**120**) exhibited an excellent activity toward p38 MAP kinase (IC_50_ = 60 nM) and an improved cell activity (dWBA IC_50_ = 48 nM) which was almost equivalent to the measured enzymatic activity.

**Scheme 23:**
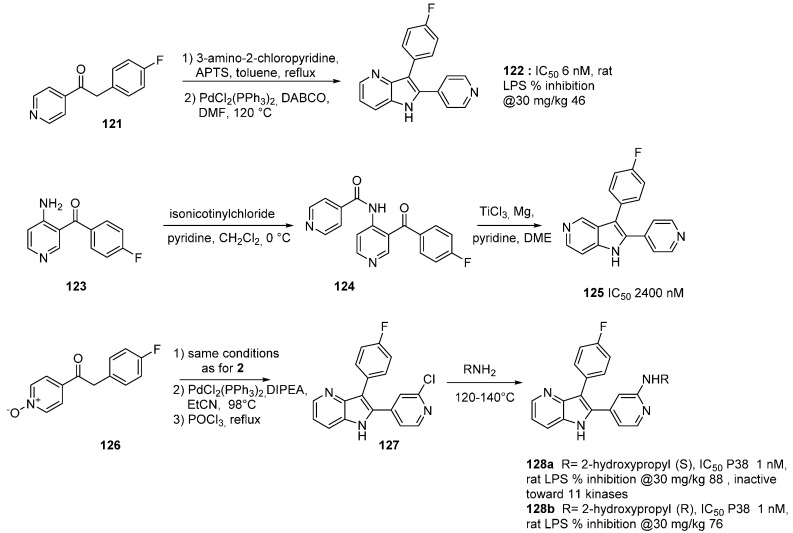
Synthesis of MAP kinase inhibitors.

In another example, the 4- or 5-azaindole derivatives **122** and **125** were described [97]. These compounds optimally interacted with the binding site through a pyridyl moiety (H-bond with Met 109), and the 4-fluorophenyl substituent which filled a hydrophobic pocket (Thr 106). The 4-nitrogen atom of the 4-azaindole was the only one that hydrogen bonded with the terminal nitrogen of Lys 53 and thus considerably improved the inhibitory potential of 4-azaindoles *vs.* 5-azaindoles. The synthesis of these derivatives was performed by building the substituted azaindole core (Scheme 23). Ketone (**121**) was converted to a mixture of imine-enamine via condensation with 3-amino-2-chloropyridine in acidic media which afford the desired indole **122** after an intramolecular Heck reaction in very high yield. The approach for the 5-azaindole **125** involved the 4-aminoyridine **123** which was first reacted with isonicotinoyl chloride prior to being subjected to a tandem Fürstner reductive coupling with TiCl_3_/Mg to yield the desired 5-azaindole **125**. Modification of the *C*-2 pyridine ring prompted the authors to start with the N-oxide **126**. After cross coupling reaction, treatment with POCl_3_ led to the chloro derivative **127** which was easily subjected to nucleophilic substitution with primary amines. In the **128** sub-family, chiral derivatives **128a** and **128b** were identified for development. The *S* isomer **128a** was chosen to pursue this objective as biological and physical data as well as *in vivo* profiles were more adapted than for **128b**.

## 14. PIM Kinase Inhibitors

**Scheme 24:**
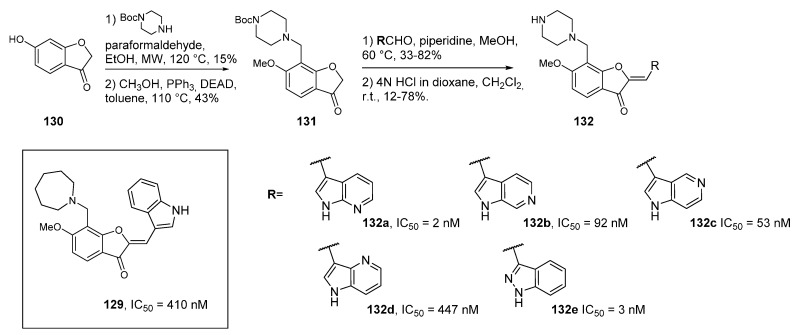
Synthesis of PIM kinase inhibitors.

PIM1 kinase overexpression in patients with hematopoietic and prostate cancer is a validated target in oncology. PIM1 phosphorylates several proteins resulting in cell survival, proliferation and migration. Enforced expression of PIM1 leads to inhibition of apoptosis and an increase in cell proliferation. In the search for novel PIM1 kinase inhibitors [99,100], an initial screening lead to compound **129** which, while showing poor metabolic properties, served as a starting point to improve potency and selectivity. A wide range of analogs were synthesized, and among these several azaindole derivatives were prepared via an aldolization reaction of the benzofuran-3-one derivative **130** with four 3-formyl azaindoles (Scheme 24). A final deprotection step afforded compounds **132**. The 7-aza derivative **132a** was the most potent PIM1 kinase inhibitor (IC_50_ = 2 nM). The lead PIM kinase inhibitor of the investigated series was compound **132e** substituted by the 2-azaindole (indazole) scaffold. Compounds **129** and **132e** showed significantly different kinase selectivity profiles and binding modes. The poorly selective compound **129** inhibited FLT3 and PDGFRα as well as the serine/threonine kinases DAPK1, GSK3β, PKCα, PKD2, and ROCK-1. In contrast, (**132e**) significantly inhibited only FLT3 (IC_50_ = 47 nM) in addition to PIM1. Selectivity within the PIM Kinase family was also observed with (**132e**) showing a high kinase selectivity towards PIM1 (IC_50_ = 3 nM) and PIM3 (IC_50_ = 13 nM) compared to PIM2 (IC_50_ = 1160 nM). This compound also inhibited the growth of the human leukemia cell line MV4-11.

## 15. PI3 Kinase Inhibitors

**Scheme 25:**
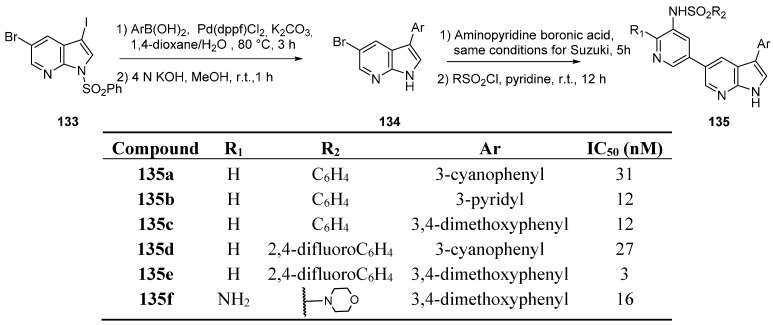
Synthesis of PI3Kα inhibitors.

Phosphoinositide 3-kinases (PI3K) are lipid kinases that catalyze phosphorylation of the 3-hydroxyl position of PIP2 to PIP3 (phosphatidylinositol 3,4,5-triphosphate) and regulate multiple physiological processes, including cell growth, differentiation, survival, and motility. Deregulation of PI3K pathway PI3K/AkT/mTOR leads to elevated PIP3 levels and downstream activation of Akt (directly or indirectly), which might be involved in the pathology of cancer, inflammation, immune disorders, and cardiovascular diseases. In particular, the PI3Kα isoform that encodes p110α catalytic subunit is implicated in a range of primary cancers [101,102,103,104,105,106,107]. A pharmacophore-directed, fragment-based strategy was reported to be the source of a drug discovery program with the use of a 7-azaindole scaffold for PI3K hinge region binding [108]. In order to find a new structural class of inhibitors giving access to the back pocket (DFG-motif, gatekeeper and catalytic lysine), the approach prioritized the incorporation of the pyridyl sulfonamide in the *C*-5 position of the azaindole. A library of final derivatives was built, starting from the benzene sulfonyl protected 3-iodo-5-bromo-7-azaindole (**133**) (Scheme 25). A palladium catalyzed arylation was first performed in the *C*-3 position whereas the second Suzuki cross coupling reaction introduced the desired *C*-5 aminopyridine scaffold group after protecting group removal. Final sulfonylation of the arylaminogroup led to increased diversity. Interesting results were obtained with compounds of type **135** which inhibited the PI3K/AKT/m-Tor pathway in the nanomolar range. Indeed **135e** exhibited the best activity against the kinase, and **135f** was selected for further evaluation because of increased solubility. Cell studies showed that this compound possessed apoptosis and anti-angiogenesis properties in hepatocellular carcinoma cells (HCC) and effectively suppressed the phosphorylation of PI3K downstream factors (AKT, m-Tor, …). The reduction of tumor vascularization was observed *in vivo*, making this compound a potential candidate for further development against human hepatocellular carcinoma.

## 16. B-Raf Kinase Inhibitors

**Scheme 26:**
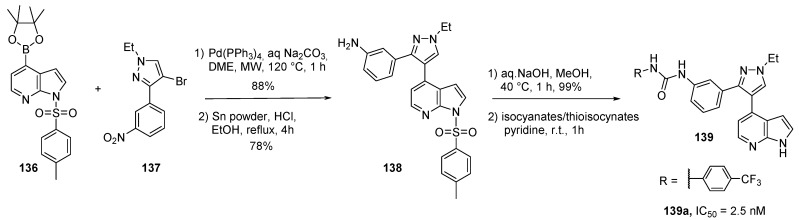
Synthesis of B-Raf inhibitors **139**.

Raf kinases play an important role in activating MEK and promoting cell proliferation and survival. The rationale for targeting the Raf-MEK-ERK signaling pathway is classically exemplified by the activity of Sorafenib (Nexavar, BAY-43-9006) which was approved in 2005 for use in renal cell carcinoma treatment and inhibits VEGFR, PDGFR, c-KIT, C-Raf and B-Raf. However, multiple preclinical animal models and clinical trials have shown mixed results using Sorafenib and developing selective chemotherapeutics acting on this kinase remains interesting. Our group has previously reported the synthesis of 4-azaindoles derivatives which target DYRK1A as well as B-Raf kinase in the sub nanomolar range (see Scheme 15) [77]. The use of a 7-azaindole scaffold in the search for a novel series of selective B-Raf inhibitors has also been described in the literature [109]. A 2-D pharmacophore map depicting the seven critical binding regions of the ATP-binding domain was used for the design of a new 7-azaindole series by incorporating functional groups that could interact with the key features of these regions. The azaindole boronic acid **136** was thus reacted with the bromo imidazole derivative **137** producing the cross-coupling derivative in good yield (88%) followed by reduction of the nitro group in the presence of tin **138** (Scheme 26). Introduction of the urea group with substituted phenylisocyanates gave compounds **139**. A thiourea analog was also prepared but was less active. Substitution in position 3 of the azaindole ring decreased activity and the *C*-2 unsubstituted 7-azaindole derivatives remained the best candidates. The best compound was the 4-trifluoro-methylphenyl substituted urea **139a** with an IC_50_ of 2.5 nM. It also showed more than a 1000-fold selectivity against a large panel of other kinases, and an almost 100-fold selectivity against VEGFR2 and c-Met.

## 17. Rho Kinase (ROCK) Inhibitors

**Scheme 27:**
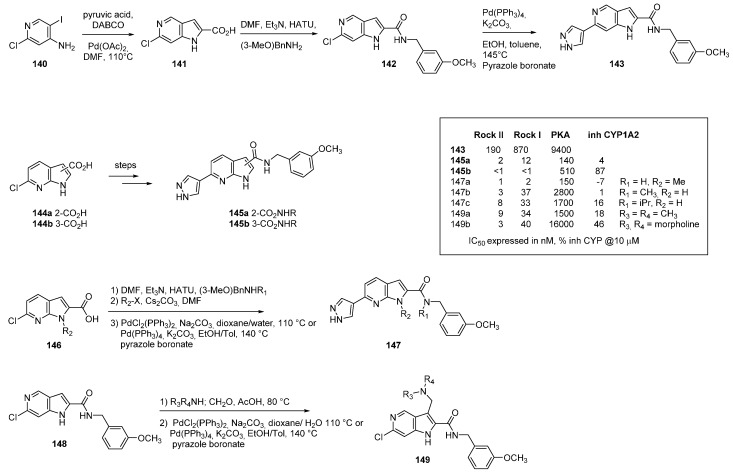
Synthesis of Rho kinase inhibitors.

Rho kinase (ROCK) is a serine/threonine kinase which plays a fundamental role in signal transduction pathways and is involved in cellular contraction, adhesion and migration. Activated by the GTP-bound G-protein Rho, ROCK phosphorylates multiple substrates. Inhibition of ROCK could therefore lead to therapeutic solutions for hypertension, glaucoma, multiple sclerosis, stroke, asthma, erectile dysfunction as well as central nervous system disorders and tumor metastasis. In two consecutive articles aimed at finding effective Rho Kinase inhibitors, [110,111] several different azaindoles were prepared (Scheme 27). An initial 5-azaindole framework **141** was built from 2-chloro-4-amino-5-iodopyridine (**140**) by reaction with pyruvic acid in a palladium catalyzed intramolecular Heck reaction. Transformation of the carboxylic acid of **141** to an amide was achieved with 3-methoxybenzylamine using standard peptide coupling to give **142**. Introduction of a pyrazole moiety at position *C*-6 was performed by a classical Suzuki cross coupling procedure **143**. This strategy was transposed to the 7-azaindole series. Synthesis of the analogous 3-carboxamide was also possible starting directly from 6-chloro-7-azaindole (prepared in two steps from commercially available 7-azaindole). Replacing the pyrazole moiety with several different heterocycles led to a decrease in activity. Discrimination of the two Rock I and II kinases is in general difficult to do and further selectivity of the best derivatives appeared using PKA activity.

Additionally it was shown that the human microsomal stability was greatly reduced by moving the carboxamide from the *C*-2 to the *C*-3 position and that the 7-azaindole scaffold is much cleaner than the indole scaffold in terms of CYP-450 enzyme inhibition. In the second article the authors further modified key derivatives by suppression of one or two amide/7-azaindole hydrogen atoms **147** and by substitution in *C-2* and *C-3* of the 7-azaindole **149**. Selectivity toward PKA was considerably enhanced, and all of the active compounds (IC_50_ < 20 nM) possessed good cellular permeability. The most promising compounds had suitable pharmacokinetic properties (DMPK) warranting potential development of the series.

**Figure 1:**
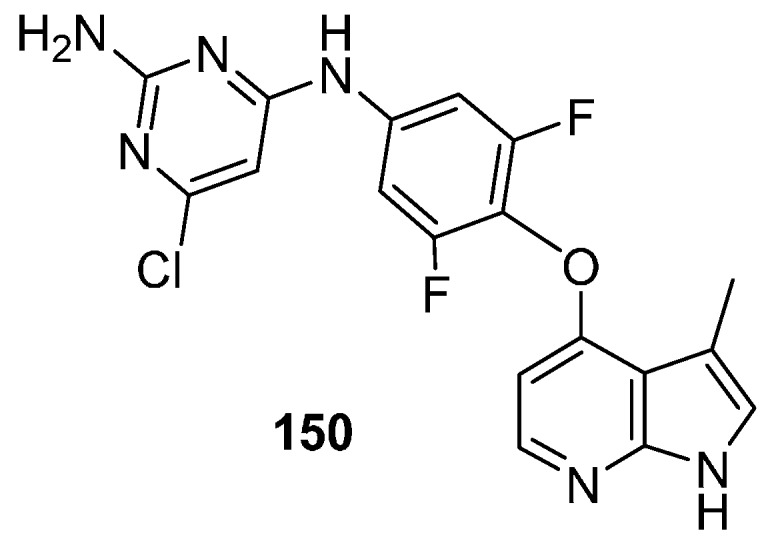
Structure of ROCK inhibitor **150**.

In 2010, workers at Bayer Schering Pharma AD published an industrial synthesis of the selective ROCK kinase inhibitor **150** (Figure 1) [112,113]. The improved synthesis gave the desired molecule (**150**) in 11 steps and 8.2% overall yield (compared to 15 steps in 0.8% yield in the first synthesis) with the unusual use of a trifluoromethyl group as a masked methyl group. This compound functions as an ATP-competitive inhibitor which acts in the sub nanomolar range for human ROCK-1 (IC_50_ = 0.6 nM) and ROCK-2 (IC_50_ = 1.1 nM). It has also been shown to induce vasorelaxation *in vitro* as well as in several *in vivo* models [114] with activity in experimental pulmonary hypertension [115].

## 18. m-TOR Kinase Inhibitors

m-Tor (mammalian target of rapamycin) belongs to a family of unconventional high molecular weight serine/threonine protein kinases. It is strongly implicated in the PI3K signaling pathway and is frequently hyperactive in human cancer [116]. mTOR is the catalytic subunit of two distinct complexes, called mTOR Complex 1 (mTORC1) and 2 (mTORC2). Inhibition of mTORC1 alone can block a desirable negative feedback mechanism, causing an increase of PI3K–Akt signaling and reduced inhibitor effectiveness. This negative feedback mechanism can be restored by inhibiting mTORC2. These observations have thus led to the search for small molecule ATP-competitive inhibitors of mTOR. The situation is further complicated by the strong structural analogy between PI3K and mTor at their ATP binding sites which has led to dual inhibitors which prevent pathway reactivation. In this context, the discovery of selective inhibitors still remains a challenging.

**Scheme 28:**
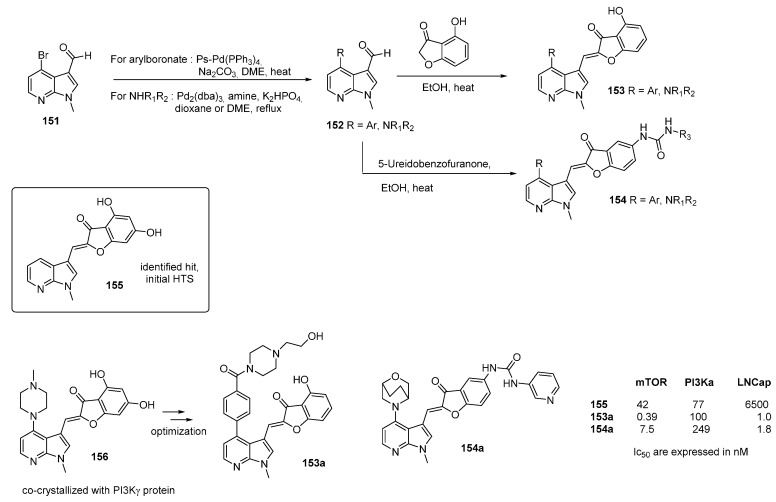
Various m-Tor inhibitors.

In the search for new structures which were both potent and selective as ATP-competitive inhibitors of mTOR, the synthesis of a two new families of 4-substituted-7-azaindoles were described (Scheme 28) [117]. Initial high throughput screening lead to the identification of an indole-bearing 4,6-dihydroxybenzofuranone **155** whose activity was increased by substituting a 7-azaindole core. In the first series **153**, the *C-4* position of the 7-azaindole scaffold was subject to intense modifications. Elimination of one hydroxyl phenol group appeared as a solution to optimize activity *vs.* selectivity parameters on enzymes, cell activity and metabolic stability. A 3D hypothesis of the binding mode was fully exemplified by docking studies, and was validated by crystallographic data.

The second study began from the metabolically unsTable 7-azaindole polyphenol derivative **156** [118]. Co-crystallization of this structure with PI3Kγ lead to the synthesis of another series of molecules **154** in which the phenolic hydroxyl groups were replaced with an ureido group, a known isostere. A large library of compounds was thus produced. In the **154** series, the position of urea attachment and the nature of the urea residues were modified. As postulated, an ureido group was metabolically better than two phenolic hydroxyl groups. Derivative **154a** appeared as the most promising derivative of the series. Access to both compounds was achieved by an aldolization reaction of a substituted benzofuran-3-one with the corresponding 3-formyl-7-azaindole **152**.

**Scheme 29:**
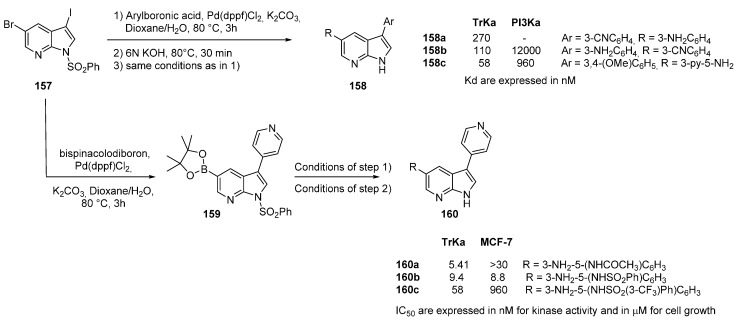
Synthesis of TrkA kinase inhibitors.

## 19. TrkA Kinase Inhibitors

The Trk sub family is composed of three receptor tyrosine kinases. Tropomyosin-related kinase A (TrkA) is involved in malignant transformation, metastasis, survival, migration, angiogenesis and invasion signaling in cancers. Additionally a defective TrkA signaling diminishes pain perception. Very recently, the 7-azaindole framework was reported in the literature in the design, synthesis, and evaluation of Trk inhibitors in cancer and angiogenesis [119]. The library was produced as previously described for ALK inhibitors (see Scheme 1), or via the construction of a 7-azaindole common intermediate **159** which was used to introduce molecular diversity in *C*-5 (Scheme 29). Affinities measured against TrK were compared to those obtained with PI3K. 7-Azaindoles bearing methoxyphenyl, cyanophenyl, and amino phenyl groups in *C*-3 and 3-aminated (Het)Ar groups in *C-5* were designed. Improved selectivity was observed with compounds of type **160** with a 4-pyridyl moiety. Further variation of the substituent in position *C*-5 gave compound **160c** as the best derivative in this series which was further subjected to kinase selectivity profiling over a panel of 30 cancer related kinase. Both cell growth and intra cell kinase activity were measured. Docking results showed that the nitrogen atom of the pyridine formed an H-bond with Lys544, the NH of the 7-azaindole donated an H-bond to Glu590, and the 7-N formed an H-bond with Met592. This strong apoptotic drug was also shown to possess anti-angiogenic effects by inhibiting cell migration and microtubule formation.

## 20. Azaindole Binding Mode Analysis

The use of X-ray crystallography in kinase research gives structural insights necessary to design more potent and selective inhibitors. A substructure search in the updated MOE kinase database [120] and in the RCSB Protein Data Bank [121] using smiles and the keyword “kinase” provided a total of 94 entries. Molecules containing azaindoles within a tricyclic structure were then removed. Only crystal structures containing protein kinase domains as described by Manning and co-workers [122] were kept. After applying all the above mentioned filters, a final total of 58 crystal structures containing 3, 7 and 48 entries for 4-, 5- and 7-azaindole frameworks respectively were obtained (Table 4). Interestingly, no crystal structure of 6-azaindole was present in the crystallographic database except tricyclic-included 6-azaindole ligands.

**Figure 2:**
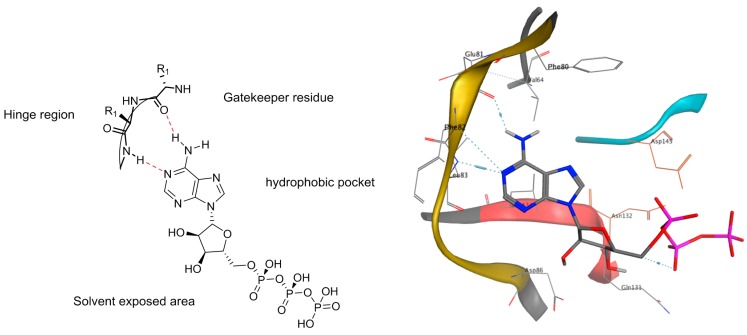
Binding mode representation of ATP in the schematic active site (Left) and in the CDK2 X-ray crystal structure (PDB code: 1FYN).

The natural substrate, ATP, binds to the hinge region of the protein kinase through two hydrogen bonds. As shown in Figure 2, the adenine moiety of ATP contains one hydrogen bond acceptor and one hydrogen bond donor exposed to the secondary amine and the carbonyl group from the amino acid backbone respectively [123].

**Figure 3:**
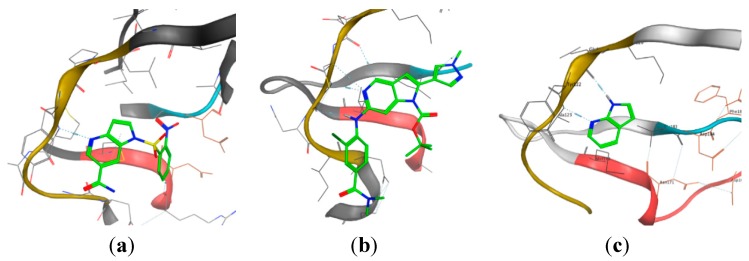
X-ray crystal structures of (**a**) 4-azaindole binding to c-Met kinase (PDB code: 2WD1); (**b**) 5-azaindole binding to TTK kinase (PDB code: 4C4J); (**c**) 7-azaindole binding to PKA-PKB chimera kinase (PDB code: 2UVX).

Over the last decades, most of the heterocyclic frameworks of kinase inhibitors have been designed to mimic the adenine moiety of ATP and therefore it’s binding to the hinge region of the protein [124]. As presented in Table 4 and Figure 3a only one crystal structure (PDB code: 2WD1) of a 4-azaindole framework binds to the hinge region [62]. The nitrogen present in the pyridine ring acts as an hydrogen bond acceptor. 

**Figure 4:**
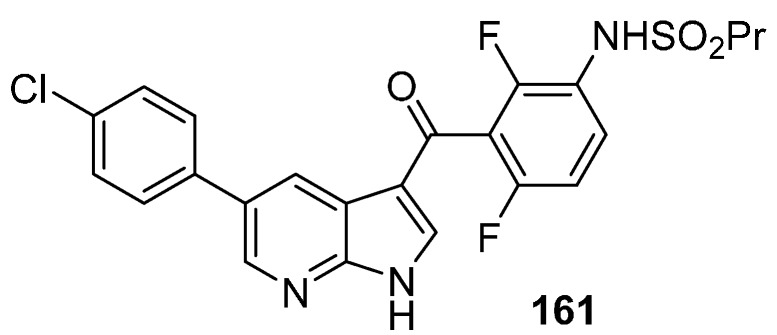
Structure of vemurafenib.

Most of the 5-azaindole-included ligands bind into the ATP active site by forming hydrogen bond interactions through the nitrogen of the pyridine and the secondary amine linked to the azaindole scaffold (Figure 3b). A medicinal chemistry strategy used in kinase research for creating hydrogen bond accepting functionality binding to the hinge region is to include a secondary amine, often a substituted aniline, linked to the heterocyclic scaffold [125]. This approach has been applied to many heterocyclic scaffolds [126]. Vemurafenib (**161**), a B-Raf (V600E) inhibitor containing a 7-azaindole framework and designed from a fragment-based drug discovery approach (Figure 4), is the only FDA approved small-molecule kinase inhibitor containing an azaindole framework [127].

Interestingly, a 7-azaindole fragment has been co-crystallized in a PKA-PKB chimera complex from a fragment-based screen (Figure 3c). This fragment occupies the ATP-binding site and perfectly mimics hydrogen bond donating/accepting functionality of the adenine of ATP through the N-H of pyrrole and N of pyridine [128]. It is therefore not surprising that this scaffold occurs more frequently in kinase inhibitors compared to 4-, 5- and 6-azaindoles, and that 48 X-ray crystal structures of ligand/protein kinase complexes contain the 7-azaindole scaffold were found (Table 4).

**Table 4:** List of crystal structures based on their binding mode.

Azaindole Binding to the Hinge Region and Mimicking the Adenine of ATP	4-Azaindole	5-Azaindole	6-Azaindole	7-Azaindole
Yes	2WD1	4C4E 4C4F 4C4G 4C4H 4C4I 4C4J	None	1ZYS 2QHM 2QOH 2UVX 2Z60 3BHT 3BHU 3C4C 3C4D 3C4E 3C4F 3CE3 3CTJ 3DJ6 3DK3 3DK6 3DK7 3E87 3ETA 3FQH 3GFW 3GQL 3HDM 3HDN 3JY9 3LJ3 3LVP 3OG7 3RCJ 3ZCL 3ZLS 4AOI 4AWI 4BIC 4BID 4BIE 4FK3 4FV9 4GU6 4HVS 4HW7 4IQ6 4JOA 4K1B 4O91
No	1OZ1 3LVP	4PMS	None	2QD9 3EN4 4JG7

**Figure 5:**
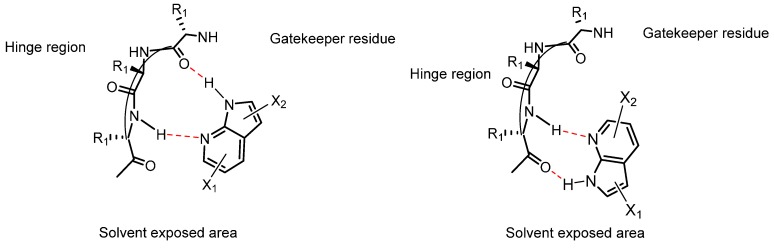
Representation of the two binding mode orientations of 7-azaindoles interacting with the backbone amides in the hinge region of the kinase.

Moreover, the 7-azaindole scaffold can bind in two different ways, both interacting through bidentate hydrogen bond donating/accepting functionality of the heterocyle to the backbone amides in the hinge region of the kinase (Figure 5). While most of the 7-azaindole-included molecules bind with the 5-membered ring pointing towards the gatekeeper residue, a few examples have the 7-azaindole moiety flipped into the active site as observed in crystal structures 3LVP, 3DJ6, 3CTJ, 3CE3, 3FQH, 3GU6 and 4AWI. Therefore, when considering 7-azaindole-containing compounds both orientations of the heterocycle into the active site have to be taken into account during the design of kinase inhibitors. Moreover, when adding a secondary amine to the pyridine ring forming the 6-amino-7-azaindole scaffold, a three-dentate hinge binder could be formed as found in crystal structure 3GFW.

## 21. Natural Products as Kinase Inhibitors

Azaindole derivatives which inhibit kinases are mainly synthetic derivatives issued from chemical creativity, HTS campaigns or *in silico* and crystallographic approaches. Nature can also be a vast source of inspiration in drug design. In this area two series will be examined.

**Scheme 30:**
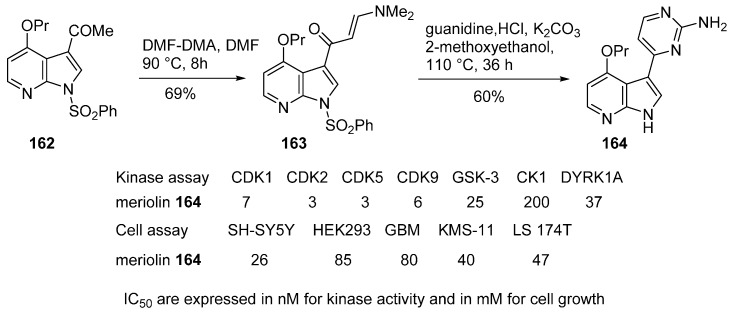
Synthesis of meriolin (**164**).

The first series is related to Meriolins which are chemical hybrids of the marine natural product families of meriadinins and variolins [129,130]. Meriolins display potent inhibitory activities toward cyclin-dependant kinases (CDKs), and are proapoptotic and antiproliferative agents. A group of 14 meriolins were synthesized and tested. Compound **164**, a representative of this series, exhibited IC_50_ values of 7 nM (CDK1) and 3 nM (CDK2), and was easily obtained in two steps from 3-acetyl-7-azaindole (**162**) (Scheme 30). The 2-aminopyrimidine substituent was sequentially introduced by reaction of DMFDMA with the methyl group of ketone **162** producing **163** in 69% yield, followed by heating with guanidine in 2-methoxyethanol to give compound **164**. This derivative is an unselective kinase inhibitor which affects several kinases in the nanomolar range, and which was co-crystallized with CDK2/cyclin A.

**Scheme 31:**
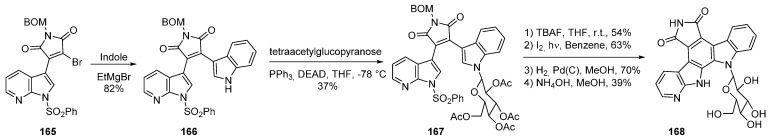
Monoazaanalogues of rebeccamycin.

In a second example, rebeccamycin, a microbial metabolite isolated from cultures of *Saccharothrix aerocolonigenes* was found to introduce breaks in eukaryotic DNA. It was also found to be a weak topoisomerase 1 inhibitor toward protein kinase C and protein kinase A. Its poor solubility however, has precluded its use as an anticancer agent. Our laboratory has been involved in the synthesis of numerous polycyclic analogs of this molecule [130,131]. A monoaza derivative was prepared from the BOM (benzyloxymethylacetal) protected azaindole bromomaleimide **165** (Scheme 31). The indole was introduced with the classical Grignard indole reagent, and glycosylation was achieved using Mitsonobu conditions **167**. After the removal of the indole protecting group, an oxidative UV cyclization followed by the sequential deprotection (hydrogenolysis, aminolysis) produced **168** in 39% yield. This derivative inhibited Chk1 in the sub micromolar range (IC_50_ = 327 nM).

**Scheme 32:**
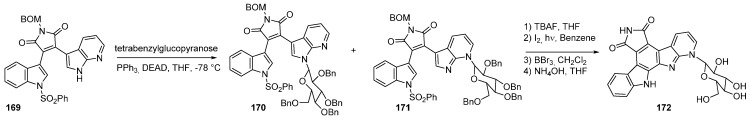
*N*7-glycosylated monoazaanalogues of rebeccamycin.

A second synthesis was elaborated to place the glucoside moiety on the 7-azaindole (Scheme 32). In this case, during the Mitsunobu reaction of **169**, the *N*1–glycosylated **170** was the major reaction product with a minor amount of *N*7-glycosylated derivative **171**. Compound **171** was then transformed into **172** using a similar approach as for **168** [132]. Chk1 inhibition increased (IC_50_ = 61 nM).

**Scheme 33:**
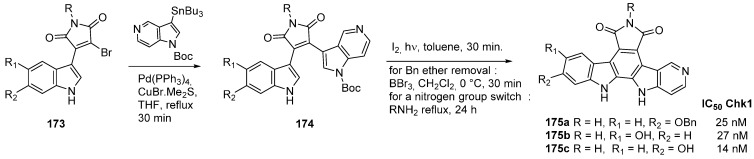
The 5-azaindole rebeccamycin series.

Similarly, a 5-azaindole series **175** was developed (Scheme 33) [29]. Synthesis was achieved using a palladium catalyzed Stille-type reaction of **173** to avoid *N*-1 instead of *C*-3 coupling of the azaindole when the Grignard reagent was employed. Photochemical cyclization, aromatization, and protecting group removal led to the original scaffold **175**. Glycosylation could be performed to mimic Rebeccamycin, and maleimide substitution could be realized by a simple amine exchange. Chk1 activity was measured and the best results occurred with only the aglycone moiety as exemplified by derivatives **175a**–**c**.

**Scheme 34:**
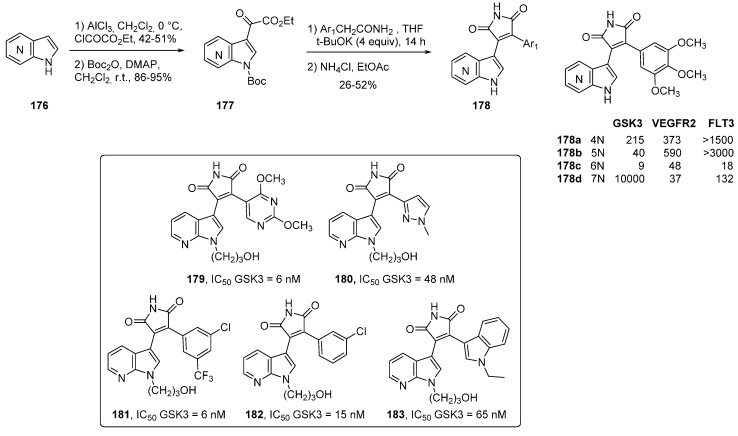
3-(7-azaindolyl)-4-(het)arylmaleimides.

Simplifying this series was possible with the functionalization of bisarylmaleimide groups, chemical intermediates of the indolocarbazoles series, which led to several strong kinase inhibitors (Scheme 34). Among these, 3-azaindolyl-4-arylmaleimides **178** were prepared according to Faul’s method from azaindole ketoesters **177**, and their biological properties were evaluated [133,134]. Significant inhibition of protein kinase such as VEGFR and GSK-3β were found. The 6-azaindole derivative **178c** with Ar_1_ = 3,4,5 trimethoxyphenyl had IC_50_ values of 48 nM for VEGRF2, and 9 nM for GSK-3β (Scheme 34). In comparison, the 7-azaindole analogue **178d** had a slightly better inhibition of VEGRF2 of 37 nM but was inactive against GSK3β. IC_50_ values for the 4- and 5-azaindole derivatives were about 10-fold higher for VEGRF2. FLT-3 kinase, which plays a crucial role in cell proliferation and differentiation, was most potently inhibited by the 6-azaindole derivative **178c** with IC_50_ values of 18 nM. Other reports complete the family, with the introduction of heterocycles instead of simple aryl groups and a hydroxyalkyl side chain on 7-azaindoles **179**–**183** which considerably increased GSK3 activity [135].

**Figure 6:**
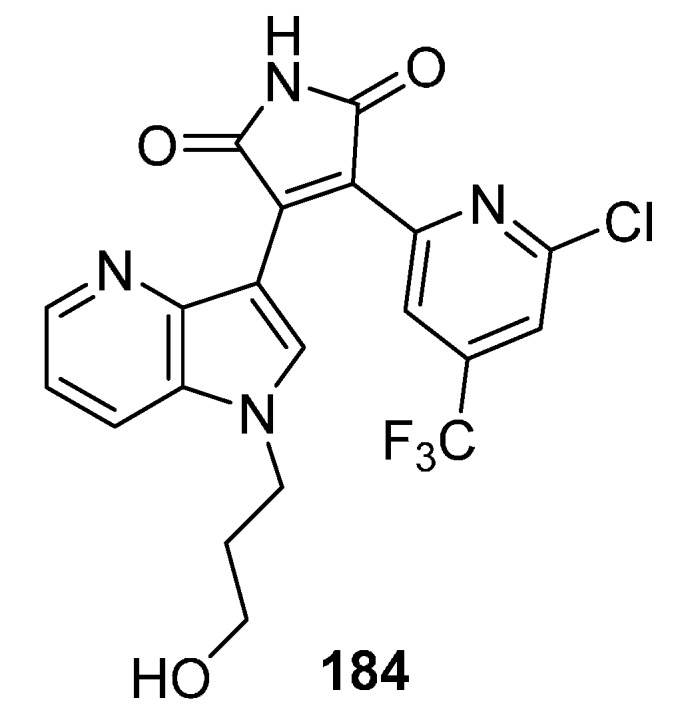
GSK-3 inhibitor **184**.

In a different study, docking of the 7-azapyridyl derivative **184** (Figure 6) in the ATP binding site of GSK3β showed several key interactions: two H-bonds between the maleimide motif to Asp133 and Val135 backbone carbonyl and amide hydrogen, respectively. The azaindole nitrogen, being only about 3.6 from the carboxyl group of Asp200 may form an additional H-bond [7]. Bisarylmaleimides, also known as moguntinones have been very recently described as new selective inhibitors for the treatment of human colorectal cancer [136].

**Scheme 35:**
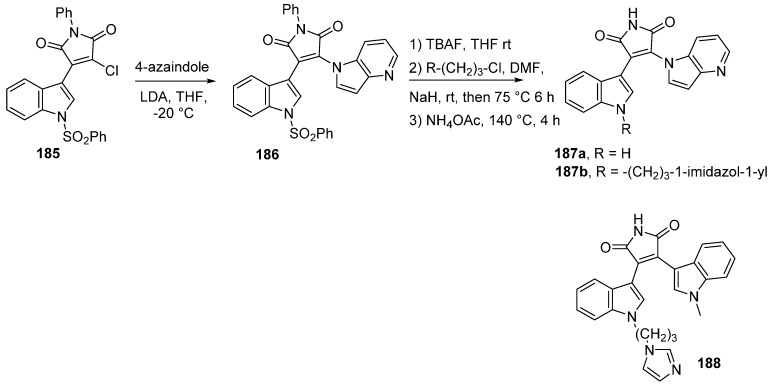
Synthesis of 4-azaindolyl–indolyl-maleimide GSK-3β inhibitors.

In a similar strategy, the synthesis of 4-azaindolyl–indolyl-maleimides lead to increased GSK-3β inhibition when the azaindole nitrogen atom was alkylated (Scheme 35) [137]. The unprotected indole derivative **187a** (R = H) had an IC_50_ value of 30.6 μM whereas compound **187b** with R = (3-imidazol-1-yl)propyl, had an IC_50_ value of 0.55 μM. Compound **188** with the same substituent on the nitrogen atom of the indole moiety had a similar IC_50_ value of 0.66 μM. Even if the activity remained in the submicromolar range, a nice selectivity for GSK-3β was observed compared to the reference staurosporine.

**Figure 7:**
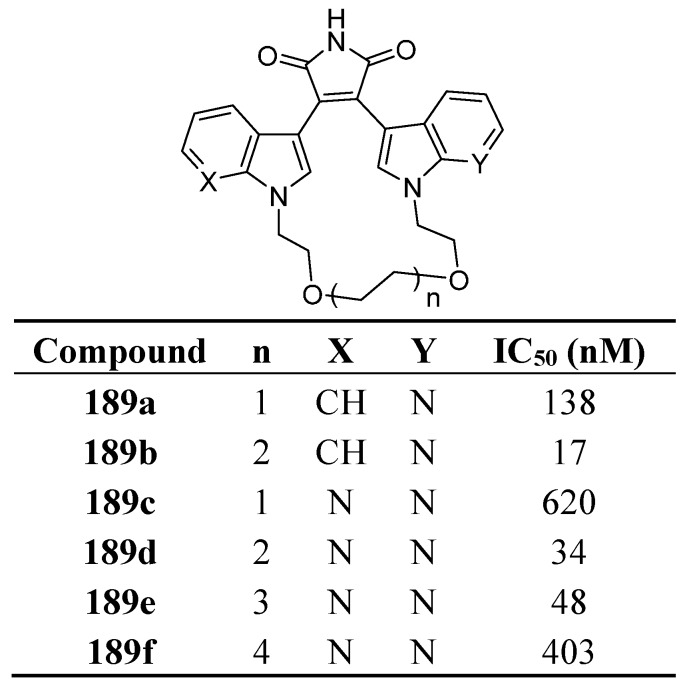
Macrocyclic polyoxygenated bis-7-azaindolylmaleimides as GSK-3β inhibitors.

In another series (Figure 7), the presence of a polyether bridge gave interesting macrocyclic structures with GSK-3β activity and an increase in selectivity [135,138].

**Scheme 36:**
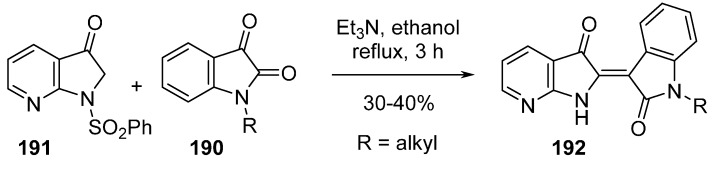
Synthesis of azaindirubins.

The next natural structure which has inspired the chemist’s imagination is indirubin (the 2,3' bis indole dimer). The natural product and its analogues inhibit cyclin-dependant kinase (CDK) but are poorly soluble [139]. Azaindirubins were synthesized in order to increase pharmacodynamic properties (Scheme 36). Aldolisation reaction by refluxing isatins **190** with 1-benzenesulfonyl-7-azaindolinone (**191**) in ethanol in the presence of triethylamine produced compound **192** as a *Z* isomer with loss of the benzenesulfonyl group. CDK2 kinase inhibitory activity was determined and compound **192** R=H having an IC_50_ value of 8.8 μM was reported, but was less potent than indirubin itself (IC_50_ = 2.4 μM).

**Figure 8:**
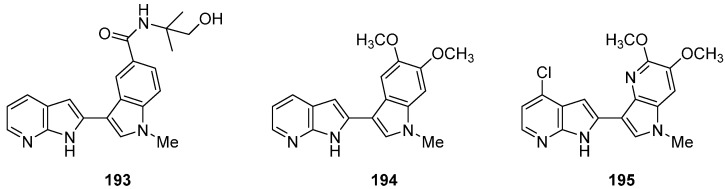
Potent C-C linked azaindole compounds.

Incorporation of a simple C-C bond between two azaindole moieties led to potent Spleen tyrosine kinase (Syk) inhibitors (Figure 8) [140,141]. This target is a cytosolic non-receptor kinase that mediates immunoreceptor signaling. Inhibition of this enzyme is an attractive strategy for the treatment of allergy, asthma and rheumatoid arthritis. Compound **193** had an IC_50_ value of 0.57 μM. Crystallographic analysis indicated that the acceptor and donor nitrogen of the 7-azaindole core forms an H-bond with the backbone NH and carbonyl of the hinge residue A451, respectively [140]. In a different series, insulin-like growth factor (IGF), which promotes growth and mediates metabolic signals, could be targeted by other bisazaindole derivatives. Agents capable of inhibiting IGF receptors would have potential as anticancer agents [142]. Compound **194** was obtained from a high throughput screen (IGF1-R IC_50_ = 55 nM), and selectivity against a panel of kinases was also evaluated. This compound was a potent Syk (IC _50_ = 55 nM) and IRK (insulin receptor kinase) inhibitor (IC_50_ = 86 nM). Activity significantly decreased with the introduction of a methyl group at the 7-azaindole nitrogen IC_50_ IGF1-R > 10 μM. Compound **195** with both a 4-azaindole and a 7-azaindole motif is the best inhibitor (IGF1-R IC_50_ = 2 nM). This increase in inhibition is the result of an active conformation where the nitrogen atom of the 4-azaindole interacts with the H3 of the 7-azaindole moiety.

**Scheme 37:**
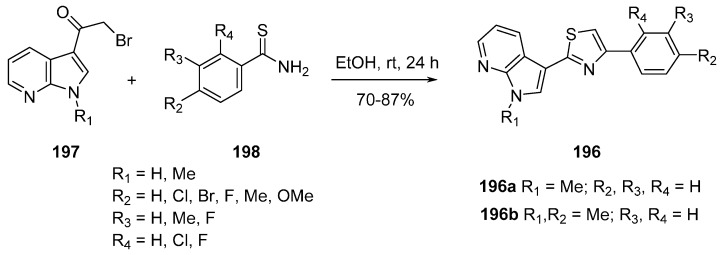
Synthesis of hybrid indolylthiazole CDK1 inhibitors.

In a last example, nortopsentins A-C, marine natural products having a characteristic 2,4-bis(3'-indolyl)imidazole skeleton have inspired the synthesis of bisindolylthiazoles which have shown strong inhibitory activity against a wide range of human cancer cell lines [143,144]. The 7-azaindole scaffold was used to create hybrid indolylthiazole structures in which one indole ring was replaced by a phenyl and/or an azaindole moiety and activity against CDK1 was tested. The synthesis of 3-(2-phenyl-1,3-thiazol-4-yl)pyrrolo[2,3-*b*]pyridine (**196**) was based on the Hantzsch reaction between α-bromoacetyl compounds **197** and thioamides **198** (Scheme 37) [145]. Two compounds **196a** and **196b** exhibited CDK1 inhibition with IC_50_ values of 0.41 and 0.85 μm respectively, but no activity was detected for CDK5 and GSK3β.

## 22. Conclusions

This review, which focuses on the synthesis and the use of azaindoles in the design of new kinase inhibitors, clearly shows that this scaffold continues to be of major importance for the preparation of biologically active compounds. The creation of an azaindole “platform”, functionalized by a variety of reactions (lithiation, protection, oxidation, palladium catalyzed coupling *etc.*) is the key to molecular diversity. These compounds display a strong biocompatibility, and are easily tolerated in living organisms. The azaindole is an excellent bioisostere for the indole ring system and this fact is confirmed by the growing number of literature references each year which use this concept to create active molecules. In parallel to the drug design process which brings together medicinal chemists, computational chemists, biologists and pharmacologists, an important number of fundamental organic syntheses are also being developed. New reactions and methodology based on the creativity of organic chemists are being explored with (hetero)-aromatic or heterocyclic non-aromatic systems (pseudo-sugars and peptides). These strategies make full use of the different possibilities offered by the growing field of organocatalysis, the use of rare metals or enzymes to remove restrictions and create new structures or functionalization. One day, some of them will undoubtedly be applied to the azaindole ring system, and thus the necessary continuum that exists between fundamental chemistry and medicinal chemistry will be reinforced.
